# Structural and Functional Annotation of Hypothetical Proteins from the Microsporidia Species *Vittaforma corneae ATCC 50505* Using *in silico* Approaches

**DOI:** 10.3390/ijms24043507

**Published:** 2023-02-09

**Authors:** Lilian Mbaisi Ang’ang’o, Jeremy Keith Herren, Özlem Tastan Bishop

**Affiliations:** 1 Research Unit in Bioinformatics (RUBi), Department of Biochemistry and Microbiology, Rhodes University, Makhanda 6140, South Africa; 2 International Centre of Insect Physiology and Ecology (icipe), Nairobi P.O. Box 30772-00100, Kenya

**Keywords:** microsporidia, unknown proteins, computational annotation, protein function prediction

## Abstract

Microsporidia are spore-forming eukaryotes that are related to fungi but have unique traits that set them apart. They have compact genomes as a result of evolutionary gene loss associated with their complete dependency on hosts for survival. Despite having a relatively small number of genes, a disproportionately high percentage of the genes in microsporidia genomes code for proteins whose functions remain unknown (hypothetical proteins—HPs). Computational annotation of HPs has become a more efficient and cost-effective alternative to experimental investigation. This research developed a robust bioinformatics annotation pipeline of HPs from *Vittaforma corneae*, a clinically important microsporidian that causes ocular infections in immunocompromised individuals. Here, we describe various steps to retrieve sequences and homologs and to carry out physicochemical characterization, protein family classification, identification of motifs and domains, protein–protein interaction network analysis, and homology modelling using a variety of online resources. Classification of protein families produced consistent findings across platforms, demonstrating the accuracy of annotation utilizing *in silico* methods. A total of 162 out of 2034 HPs were fully annotated, with the bulk of them categorized as binding proteins, enzymes, or regulatory proteins. The protein functions of several HPs from *Vittaforma corneae* were accurately inferred. This improved our understanding of microsporidian HPs despite challenges related to the obligate nature of microsporidia, the absence of fully characterized genes, and the lack of homologous genes in other systems.

## 1. Introduction

Microsporidia consist of a genus of early-diverging obligate intracellular microorganisms found infecting a variety of organisms [1,2,3]. They have an important impact on both environmental research and clinical diagnostics [1,2,4]. Microsporidia rely on their host for many metabolic processes [5,6,7,8]. Several studies demonstrate that this phylum of microorganisms has undergone significant gene loss over time, leading to their compact genome sizes that range from 2.3–52 Mbp [9,10,11,12,13,14]. Microsporidia are found in a wide range of hosts, from small invertebrates to higher vertebrates. Furthermore, they have different effects on their hosts, with some reported to be symbiotic while others are pathogenic [15,16,17,18]. Understanding the genome of microsporidia is therefore important to discern the genetic basis of different species and their unique phenotypic effects on their host [19,20].

Hypothetical proteins (HPs) are a set of proteins for which no experimental data are available for their *in vivo* expression. An investigation into the structure and function of these proteins could be used to inform their position in metabolic pathways and hence decipher their implications for the biology of these microorganisms and their infection phenotypes [21,22,23,24,25]. There are several reports on fast and accurate computational methods for annotating HPs from important human pathogens [21,26,27]. Notably, functional annotation of HPs aided in the recent discovery of novel abiotic stress proteins in *Triticum aestivum* [28]. Moreover, HP annotation can be used to identify new targets for drug design and discovery [29].

The microsporidia proteome consists of over 50% of HPs that presumably play an important role in the physiological and biochemical processes of these microorganisms [30]. This study highlights several bioinformatics approaches for predicting the structure and function of previously unassigned proteins in the *Vittaforma corneae ATCC 50505* (*Nosema corneum*) proteome. *Vittaforma corneae ATCC 50505* is a human pathogen that affects the ocular tissues of immunocompromised individuals. The species is characterized by a relatively small genome size of 3.2 Mbp, encoding a total of 2237 proteins, 2034 of which are classified as HPs [31]. It is therefore important to identify the function of these proteins to provide insight into the biology of this microorganism. 

Several online tools are currently available for reliable protein function prediction. In this study, the functional prediction was considered to be of high confidence level, where the results of three or more separate tools were in agreement. Briefly, this research describes the use of different tools in providing a robust annotation of a group of previously unknown proteins from the human pathogen *Vittaforma corneae ATCC 50505*. The identification and retrieval of similar well-characterized proteins were performed using NCBI-BLASTp [32]. Protein family classification was thereafter conducted using multiple tools including Pfam [33], InterPro [34], Protein Analysis Through Evolutionary Relationships (PANTHER) [35], PRINTS [36], Protein Information Resource Superfamily (PIRSF) [37], and GENE3D [38]. Additionally, the identification of key domains was carried out using the Conserved Domain Database (CDD) Search [39,40], PROSITE [41], and the Simple Modular Architecture Research Tool (SMART) [42]. Motif analysis was performed on MEME Suite [43] and conserved motifs were analysed to identify the close relationship between homologous characterized protein sequences. The physical and chemical properties of the retrieved HPs were predicted using the ExPASy-ProtParam tool [44]. Subcellular localization of the HPs was determined using TargetP [45], SignalP [46], TMHMM [47], and Phobius [48] online tools. Moreover, protein network analysis using the STRING database was used to identify key interacting proteins with specific HPs [49,50]. Additionally, sequence similarity comparisons between the HP and respective homologs were performed through multiple sequence alignment, sequence identity calculations, and phylogenetic analysis. Furthermore, homology modelling of the 3D structure of these HPs was predicted using PRotein Interactive MOdeling (PRIMO) [51].

Overall, this work sheds light on the different strategies available to accurately characterize the HPs. The in silico approach used in this study for the functional and structural analysis of these proteins is important to improve the understanding of the mechanism of action of the microsporidian. The research design used here was adapted from several articles that applied the same approach in different organisms [23,28,52,53,54] and were able to accurately decipher the functions of unknown proteins. We proceed a step further to demonstrate important structural and functional analyses of a few case studies from the batch of HPs identified in this species. The pipeline and the information from this study could furthermore be used in the study of other closely related microsporidians to assess the crucial interactions between the microorganism and its host. 

## 2. Results and Discussion

**Figure 1:**
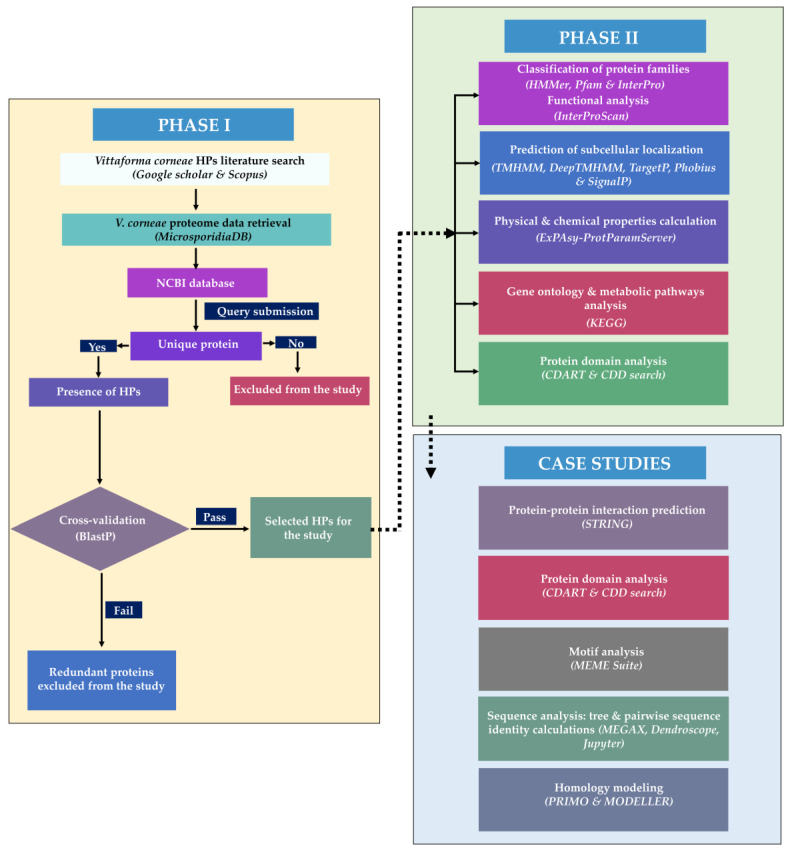
A summary of the computational approach used to annotate *Vittaforma corneae ATCC 50505* hypothetical proteins. The pipeline highlights 2 major phases: PHASE I. Sequence retrieval and similarity search. PHASE II. Diverse functional annotation and sequence analysis steps using different tools to predict protein families, localization, motif and domain identification, and chemical properties of the HPs. Additionally, sequence identity calculations, homology modelling, and phylogenetic analysis were conducted on five selected case studies.

This study has two consecutive phases (Figure 1): PHASE I involved the identification and retrieval of unique HPs from online databases and homology search. PHASE II involved the functional and structural analysis of the selected proteins including protein family classification, characterization of the physicochemical properties of the HPs, prediction of membrane proteins and presence of signal peptides, homology modelling, identification of their interacting proteins, and the metabolic pathways. A list of bioinformatics tools and resources used for the functional annotation of *Vittaforma corneae ATCC 50505* HPs is given in Supplementary Table S1.

### 2.1. PHASE I

#### 2.1.1. Sequence Retrieval

No experimental or computational analyses have been previously performed to characterize HPs present in *Vittaforma corneae ATCC 50505*. Therefore, this study aimed to perform this using in silico approaches. The *Vittaforma corneae ATCC 50505* proteome was retrieved from NCBI and consists of a total of 2237 unique protein sequences [55]. Out of these, 2034 (90.97%) of the proteins were classified as hypothetical while 203 (9.1%) were fully characterized proteins [55]. In addition, 207 of the HPs were labelled as fragmented/partial protein sequences in the FASTA file as retrieved from NCBI, while 1827 were complete protein sequences. This study focused on the nonfragmented sequences. Moreover, the retrieved protein sequences were further looked up in UniProt to determine the availability of annotation data using a batch search within the database (database accessed in December 2022).

#### 2.1.2. Sequence Similarity

A total of 627 (34.31%) of the 1827 whole HP sequences did not have any homologous sequences in the NCBI database, following a BLASTp homology search coupled with a PSI-BLAST search. Since sequence homology is a key first step to inferring protein family classification and function, any HP whose BLAST hits were other HPs were removed from the dataset. This was a total of 845 HPs (46.25%). A total of 355 (19.43%) HP sequences were found to be homologous to known and characterized proteins and were therefore focused on in this study. For the 355 proteins, a BLASTp search (with a cut-off E-value of 0.0001 and a bit-score greater than or equal to 200) revealed that there were several sequences corresponding to these proteins from different species of microsporidia. The top 10 BLASTp hits for each protein from were then taken through a similar search pipeline to confirm if the homologous HP appeared in this new search as schematically highlighted in Supplementary Figure S21. Notably, the search aimed to identify the genes most closely related to the HP; therefore, increasing or decreasing the E-value would only affect the identification of distant homologs and not the top-ranked homologs. True homologs were identified as hits from sequences where the HP appeared in the top three with high coverage and percent identity. A total of 162 HPs were retained from this search and were used for the following downstream in silico characterization processes. 

### 2.2. PHASE II

#### 2.2.1. Classification of Protein Families

Microsporidian proteomes often consist of multiple protein copies performing similar functions [9,56,57]. In this study, we performed a classification using the programs listed in Figure 1 and Supplementary Table S1. Classified proteins were grouped as enzymes, DNA/RNA/protein/metal-ion binding, cellular proteins, transport proteins, or transcription factors based on their most statistically significant hit families. This classification was primarily based on Pfam annotation which infers protein family classification by identifying functional regions and domains in the query sequence through hidden Markov models (HMMs) and multiple sequence alignments [33]. A batch search of the protein sequences in question was performed through the HMMER suite with default significance E-values used set at model (query sequence) = 0.01 and hit E-value = 0.03. HMMER’s per-sequence E-value is similar to BLAST and is a measure of statistical significance that sequence hits are homologous to the HP [58]. Hit E-values, on the other hand, are used to rank the domains with the highest scores within a sequence, suggesting that domains identified in the HP may be similar to identified hit domains that guide protein family classification.

**Figure 2:**
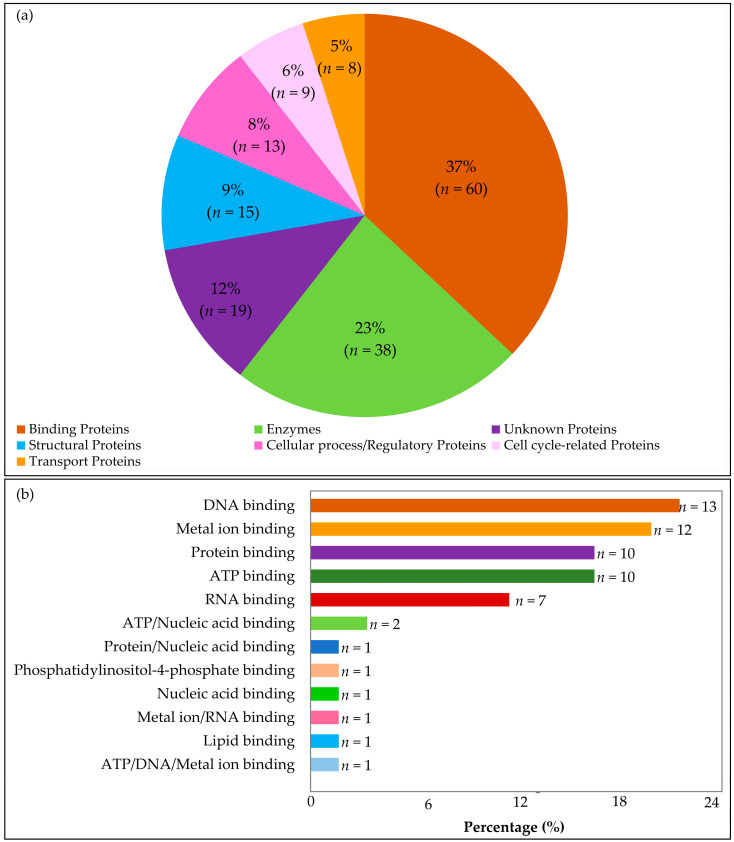
Protein family classification. (**a**) A total of 143 out of 1304 HPs classified into respective functional groups based on functional annotations using several online tools. The total number of HPs found in each group is illustrated by the number following each group name and followed by their respective percentage in brackets. The chart shows that the majority of the proteins (37%) are involved in binding to different molecules (nucleic acids, proteins, ATP), followed by enzymes (23%), structural proteins (9%), regulatory proteins (8%), cell-cycle proteins (6%), and transport proteins (5%). Cellular and regulatory proteins include those involved in translation, transcription, and replication. The unknown proteins highlighted include those that did not have significant hits across the tools used. (**b**) A bar graph representation of the proportion of binding proteins. Nucleic-acid-binding proteins, ATP, and protein-binding proteins. The numbers next to the labels indicate the total number of HPs in each group out of the total 60 binding proteins, with percentages. The order of each section is sorted by size, with the largest proportion of the binding HPs binding to DNA (13.22%) followed by metal-ion-binding proteins (12.2%).

In total, 19 of the 162 HPs were not classified. However, for a large proportion of the HPs (*n* = 143, 88.27%), a significant protein family classification hit was generated. Out of the classified HPs, the largest proportion were binding proteins (*n* = 60, 37%) followed by enzymes (*n* = 38), proteins involved in cellular processes (*n* = 13), and transport proteins (*n* = 8) (Figure 2a). Moreover, the binding proteins were further classified based on the molecule they bind to, including nucleic acids, proteins, ATP, and other substances (Figure 2b). Supplementary Tables S2 and S3 highlight the summary of the functional groups in the annotated proteins.

##### Enzymes

Enzymes are essential for catalysing metabolic processes. Reports indicate that microsporidia have a wide range of enzymes that are secreted into the host cells to control host metabolism [59]. A total of 38 HPs (23%) were predicted to be enzymes. These were further grouped into varying enzyme types including transferases, kinases, hydrolases, reductases, phosphatases, and proteinases (Table S2).

*a.*  *Transferases*

This group of proteins plays an integral role in oxidative stress. Ten Vittaforma corneae ATCC 50505 HPs were classified as transferases. XP_007603549.1 classified as S-adenosyl-L-methionine-dependent methyltransferase (SAM_MT_RSMB_NOP domain) from the RSMB superfamily is responsible for RNA methylation and detoxifying cytotoxicity. Protein XP_007604313.1 was also predicted to show transferase activity, predicted to be histone acetyltransferase. The enzyme catalyses the transcription process. This protein contains the Acyl-CoA N-acyltransferases (Nat) domain and is localized in the nucleus.

*b.*  *Kinases*

These are receptor proteins and play an important role in signal transduction and cell cycle regulation [60,61]. Two HPs were predicted to be kinases (HP XP_007603954.1 and HP XP_007604292.1). The former is predicted to be a cytoplasmic protein that contains an NAD(P)HX binding site and is involved in ADP-dependent NAD(P)H-hydrate dehydratase activity and ATP binding. Moreover, it displays conserved ATP and Mg^2+^ binding sites and is therefore classified as belonging to the ribokinase-like superfamily. On the other hand, XP_007604292.1 is also known as the TNase-like domain-containing membranous protein. It also contains the staphylococcal nuclease (SNase) homologs with an OB-fold (open beta-barrel) that mediates DNA-binding.

*c.*  *Hydrolases*

Hydrolases are a vital component in host tissue invasion and evasion of the host defence mechanisms. In this study, six HPs were predicted to be hydrolases. Four of these were classified as P-loop-containing nucleoside triphosphate hydrolases. These were XP_007604316.1, XP_007605048.1, XP_007605128.1, XP_007603893.1, and XP_007605132.1.

*d.*  *Reductase*

One HP, XP_007604630.1, was classified as dihydrofolate reductase (DHFR) containing a DHFR domain and involved in glycine and tetrahydrofolate biosynthetic processes by regulating levels of folate coenzymes.

*e.*  *Phosphatase*

Microbes secreting phosphatase deplete the host’s phosphate levels and this is one of the key factors that increase the virulence of disease-causing microorganisms. One HP was grouped as alkaline phosphatase. The protein XP_007605421.1 is involved in transferring phosphorus-containing groups. It contains eight transmembrane helices. 

##### Binding Proteins

Sixty binding proteins were predicted and classified based on the molecule they bind to, either nucleic acids, proteins, metals, ATP, or other molecules (Figure 2b).

*a.*  *Nucleic-acid-binding Proteins*

Thirteen DNA-binding and eight RNA-binding HPs were identified. Four homeobox-domain-containing HPs were identified (VICG01256, VICG01265, VICG01568, and VICG02118) and are involved in DNA-binding and transcription regulation. The histone-domain-containing DNA-binding protein VICG00524 is involved in the regulation of protein heterodimerization activity.

*b.*  *Protein-binding Proteins*

Eleven HPs were found to be members of the protein binding group. They were characterized as either containing WD-repeats domains, PFU domains, or TPR domains. Ubiquitins are globular proteins and bind to other proteins, altering their function and location. A large set of ubiquitin fusion degradation proteins were identified. They function mainly in post-translational modification and protein turnover, and also act as chaperones. The XP_007604246.1 belongs to the ubiquitin conjugation factor E4 family containing the U-box domain and is involved in ubiquitin–ubiquitin ligase activity [62].

*c.*  *Metal-binding Proteins*

Metal- and metal-ion-binding proteins take part in myriad cellular processes during DNA replication. Here, we highlight 13 metal-binding HPs. Ten of these HPs contain the zinc finger domain. Zinc-ion-binding proteins such as HP, XP_007605126.1, contain a Cys(2)His(2) (C_2_H_2_)-type domain found in transcription factors in eukaryotes. Moreover, they are also shown to be versatile in binding to other molecules such as DNA and other proteins [63]. Most importantly, the presence of zinc-binding sites in these proteins improves their stability and structural integrity in different cellular environments [64].

The Hop1p, Rev7p, and MAD2 (HORMA)-domain-containing HP XP_007605437.1 is another metal-ion-binding protein annotated in this study and is predicted to be involved in DNA repair [65]. 

##### Cellular/Regulatory Proteins

Cellular proteins are involved in important cellular processes such as translation, transcription, replication, and cell-cycle regulation. A total of 27 HPs were grouped into this category. These included nine transcription factors and transcription regulatory proteins, eight polymerases, five translation proteins, and five ribosomal biogenesis regulatory proteins. 

##### Transport Proteins

The microsporidia genome encodes numerous transporter proteins used to acquire host nutrients to support their development [66]. Transport proteins such as carriers, transporters, receptors, and signal transduction proteins play an integral role in the survival of intracellular organisms by transporting useful nutrients and metabolic waste into and out of the microorganism’s cells. 

This analysis predicted transporters, carriers, receptors, and signal transduction proteins. Notably, four ATP-Binding Cassettes (ABC) transporter proteins were predicted. ABC proteins are important in regulating the entry and exit of various substrates and the attachment of microbes onto the host cells’ surface [67,68,69]. They are characterized by a transmembrane domain at the N-terminal involved in pore formation in the inner membrane and a P-loop containing the nucleoside triphosphate hydrolase domain at the C-terminal that is involved in ATP-binding and, hence, energy generation [68]. These predicted HPs included XP_007603484.1, XP_007604934.1, XP_007603628.1, and XP_007605468.1. 

Signal transduction is a biochemical process by which a cell communicates with extracellular messenger molecules to regulate important metabolic pathways and control the growth of intracellular microorganisms. The microsporidia proteome is enriched with protein domains involved in protein–protein interactions such as signal transduction and WD40 domains [70]. This is an essential property of the obligatory intracellular behaviour of this group of microorganisms. Five HPs were predicted to be signal transduction proteins (XP_007603813.1, XP_007604255.1, XP_007604458.1, XP_007604530.1, and XP_007605241). Furthermore, the conserved domain analysis showed that these proteins also contained seven repeats of the WD40 domain which mediates the protein–protein interaction system [71,72]. 

Receptor proteins are similarly important in cell signalling by binding to signalling molecules and ligands to regulate cellular response, thereby promoting intracellular trafficking of nutrients. Two receptor proteins were annotated from the HPs. These were XP_007605437.1 and XP_007605458.1. The HP XP_007605437.1 was further characterized as containing leucine-rich repeats. XP_007605458.1 is grouped in the PHD finger superfamily containing a nuclear-receptor-binding SET domain and was predicted to be involved in controlling gene transcription. 

Carrier proteins are another important group of host-cell-interacting molecules useful in the importation of nutrients to the intracellular parasite. They contain an N-terminal domain used to infiltrate the host cell. These proteins have a ferredoxin-containing receptor-binding domain that is used to take control of the hosts’ iron uptake pathway. The HP XP_007603620.1 contains a 2Fe–2S (iron–sulphur) cluster-binding domain that takes part in electron transfer processes. 

##### Structural Proteins

Structural proteins are crucial for the survival and pathogenesis of a microbe. They provide a protective protein surface layer against extracellular, and regulate molecular, influx from the surrounding environment [73]. Microsporidia structural proteins such as spore wall proteins also function to adhere to host cells during infection [74]. Here, 16 HPs were reported to be structural proteins Table S2. XP_007604178.1 belongs to the microsporidian spore wall protein family (MICSWaP superfamily). It contains a signal peptide at the N-terminal and a noncytoplasmic domain. Table S3 further highlights these different functional groups. 

The HP XP_007604054.1 was classified as an actin-related protein, a structural constituent of the ribosome linked to the protein’s 3D structure. This protein is also associated with the nucleotide-binding domain of the sugar kinase/HSP70/actin superfamily. As such, actin is involved in filament formation, a major component of the cytoskeleton that interacts with myosin during cell motility [75,76].

#### 2.2.2. Subcellular Localization

The localization of a protein inside a cell is often linked to the respective protein’s function. Membrane-spanning proteins are reported to be involved in signalling pathways and the transportation of nutrients across different biological environments within and outside the cell [77]. For this reason, it is important to identify the presence of signal peptides and transmembrane helices within the HPs. Analysis of subcellular localization of HPs using TMHMM, DeepTMHMM, TargetP, SignalP, and Phobius identified HPs as membranous in at least three of the tools. Table S4 highlights the prediction of transmembrane helices and signal peptides using different tools, and consistent outputs are highlighted in red. DeepTMHMM analysis predicted a total of 20 transmembrane proteins, 15 signal peptides, 125 globular, and 4 HPs spanning both the transmembrane and signal peptide regions (Table S4).

More importantly, these results were compared with the GRAVY index score of hydropathy. Membrane proteins are often hydrophobic and, as such, HPs containing transmembrane helices would be considered to fall under this category, with GRAVY scores of more than zero. On the other hand, globular proteins are localized within the cell and would further be classified as hydrophilic. This analysis displays the same pattern, with 30 HPs assessed to be hydrophobic, 15 of which were also predicted to be localized on the membrane by different localization prediction tools and 5 containing signal peptides (Table S4). Out of the 30 hydrophobic HPs, 5 were predicted to have signal peptides in both TargetP and SignalP, while 6, 20, and 18 of these HPs with signal peptides were identified with Phobius, TMHMM, and DeepTMHMM, respectively.

The TMHMM tool predicts the number of transmembrane helices in proteins and highlights the expected number of amino acids in the identified helices. If the expected number is higher than 18, then the probability of the query protein being transmembrane or having a signal peptide is also high. Moreover, transmembrane helices in the N-terminal are more likely to be signal peptides. However, TMHMM cannot be used to identify if the protein is cytoplasmic, unlike other tools. 

The Phobius tool, on the other hand, predicts if a protein is cytoplasmic or noncytoplasmic alongside signal peptide prediction. This tool scores the most probable location and orientation of transmembrane helices using the N-best algorithm, such as the TMHMM tool. Similarly, TargetP and SignalP identify the presence of signal peptides and the regions of the sequence that they span across. In this survey, TargetP, SignalP, and Phobius predicted a total of 15, 12, and 22 signal peptides, respectively (Table S4). All these tools used together give a more comprehensive prediction of membranous and nonmembranous proteins. 

#### 2.2.3. Protein Characterization by Physicochemical Properties

**Figure 3:**
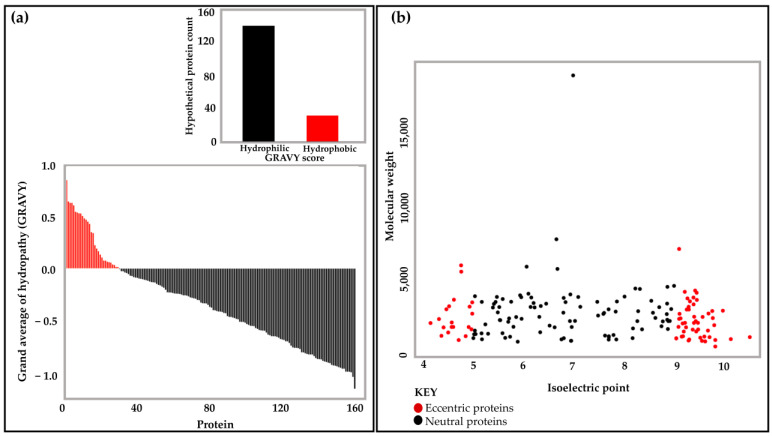
An illustration of the physicochemical properties of the annotated HPs: (**a**) The grand average of hydropathy (GRAVY) scores of less than 0 indicate the prediction of hydrophilic (globular) proteins, while scores larger than 0 are predicted to be hydrophobic (membrane) proteins. A total of 134 (81.7%) HPs were classified as hydrophilic, while 30 (18.3%) HPs were identified as hydrophobic. (**b**) Isoelectric point vs. molecular weight of HPs using the ProtParam tool. In red are the eccentric proteins with acidic pI (3–5) (*n* = 20; 12.2%) or basic pI (9–12) (*n* = 49; 29.87%), while those in black are proteins with pI range 5–9 (*n* = 95; 57.93%). The majority of HPs fall in this category.

The classification of proteins based on their physical and chemical properties is useful in identifying the stability and nature of the macromolecule for future wet-lab experiments. Physicochemical characterization is also a useful indicator of the biochemical processes that the HPs might be involved in [53,78,79]. Out of the 164 HPs, 30 (18.3%) were hydrophobic and the remaining 134 (81.7%) were hydrophilic (globular), as highlighted in Figure 3a. 

Moreover, the molecular weight and isoelectric point (pI) inform the chemical characteristics of the HPs. It was observed that a large proportion of the HPs were neutral, with pI ranging between 5 and 9 (*n* = 95, 57.93%). By comparison, the number of eccentric HPs (basic or acidic) was lower, with the proportion of basic proteins being 29.87% (*n* = 49), while that of acidic proteins was 12.2% (*n* = 20) (Figure 3b).

#### 2.2.4. Gene Ontology and Metabolic Pathway Analysis

A cross-check of the annotated HPs’ functions was carried out against the KEGG pathway database, confirming their predicted functions in different metabolic pathways (Table S5). A total of 55 HPs were accurately annotated, and this was consistent with the protein family classification (Table S3) with a large set of predicted proteins identified to be involved in catabolic and regulatory processes. 

### 2.3. Case Studies

In this section, we further zoom into some of the identified HPs and perform in-depth structural and functional analysis of these proteins. This was carried out with a combination of in silico approaches including protein–protein interaction prediction, 3D protein structure prediction, conserved domain prediction, motif analysis at sequence and structure level, and phylogenetic tree calculations. 

Our criteria for the selection of the HPs were as follows: (a) high sequence identity with homologous proteins with known functions; (b) high phylogenetic tree bootstrap values; (c) one protein per functional group; (d) proteins with different predicted functions as a representation of the functional group. A subset of five HPs (VICG00012, VICG01314, VICG01349, VICG01687, and VICG01723) was then selected to perform structural and functional analyses in comparison to their respective homologs with known functions. 

Protein function can be predicted from the analysis of neighbouring proteins. Most proteins performing similar functions will cluster together. The STRING database is a secondary platform that infers protein association networks from the information across different primary sources, including experimental sources, primary protein databases, gene neighbourhood, gene fusion, and text-mining from different publications where the query protein has been named alongside other proteins, co-occurrence, and co-expression of similar proteins. In this study, protein association networks were generated based solely on experimental evidence of known interactions. Association networks of representative HPs were analysed. Notably, the characterized proteins clustering with the annotated HPs were involved in the same biological function.

Sequence analysis methods such as CDD identify patterns and profiles (signatures) to predict conserved domains by transferring the information from experimentally characterized proteins to uncharacterized ones [40]. This makes tools such as the CDD a powerful resource for identifying superfamilies and functional and structural domains in HPs, as demonstrated in the following case studies. 

Multiple sequence alignment and phylogenetic tree analysis are useful in determining and confirming the relationships between the selected HPs and their respective homologs in different organisms. Phylogenetic studies generate evolutionary trees where species are hierarchically organized, with very closely related proteins/species grouped next to each other [80,81]. Moreover, the study of motif patterns at the sequence and structural level is useful in protein function prediction. The discovery of conserved motifs within a subset of homologous protein sequences while missing in other species would be useful in associating known protein functions with the unknown. Furthermore, the determination of repeat patterns within amino acid sequences within the same family such as leucine-rich repeats (LRR) and heparin-binding motifs aid in the protein’s structural framework and functional assessment, respectively [82,83,84,85].

#### 2.3.1. Case Study I: Functional and Structural Analysis of VICG00012

*a.*  *Protein–Protein Interaction Analysis*

The identification of a novel microsporidian polar tube protein was based on its clustering with other key proteins found in the structure of the microsporidian invasion apparatus, a key feature involved in spore germination [11,86]. 

**Figure 4:**
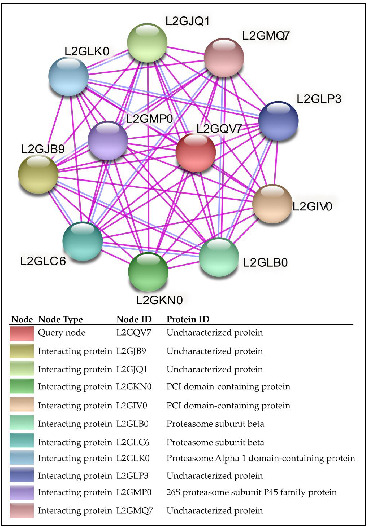
Protein–protein association network analysis using the STRING database highlights the closely interacting proteins with the query HPs. Each node represents a protein; the query node is represented in red (L2GQV7). A total of 10 interacting proteins (nodes) were observed, with a total of 55 edges. The edges indicate both functional and physical protein associations. The colour of the edges indicates the type of interaction evidence. The pink edges indicate associations from experimentally-determined associations, while the purple edges indicate protein homology. From the network analysis, the HP is shown to have close homology to a proteasome alpha 1 domain-containing protein (L2GLK0) and is experimentally linked to similar proteins. The protein–protein interaction (PPI) enrichment *p*-value is 4.66 × 10^−15^, showing that the clustered proteins in this group are at least partially biologically connected.

The HP VICG00012 (Accession number: XP_007603465.1) is classified under the family 20S proteasome alpha and beta subunits involved in posttranslational modification, protein turnover, and chaperones (Tables S2 and S3). It has close homology to proteasome subunit proteins from other microsporidia species. It is predicted to function in the proteolysis process involved in cellular catabolic processes. Its homologous superfamily is identified as nucleophile aminohydrolase (Ntn hydrolases). It is located within the proteasome core complex and contains a threonine nucleophile. VICG00012 was identified to have strong interactions with the PCI-domain-containing proteins that are characterized by α-helices and a winged-helix domain with a *p*-value of 4.66 × 10^−15^ (Figure 4). The protein was also closely associated with similar-functioning proteasome subunit proteins L2GLB0, L2GLC6, L2GLK0, and L2GMP0 from experimentally determined associations, as indicated by the pink edges (Figure 4).

*b.*  *Identification and Analysis of Conserved Motifs*

**Figure 5:**
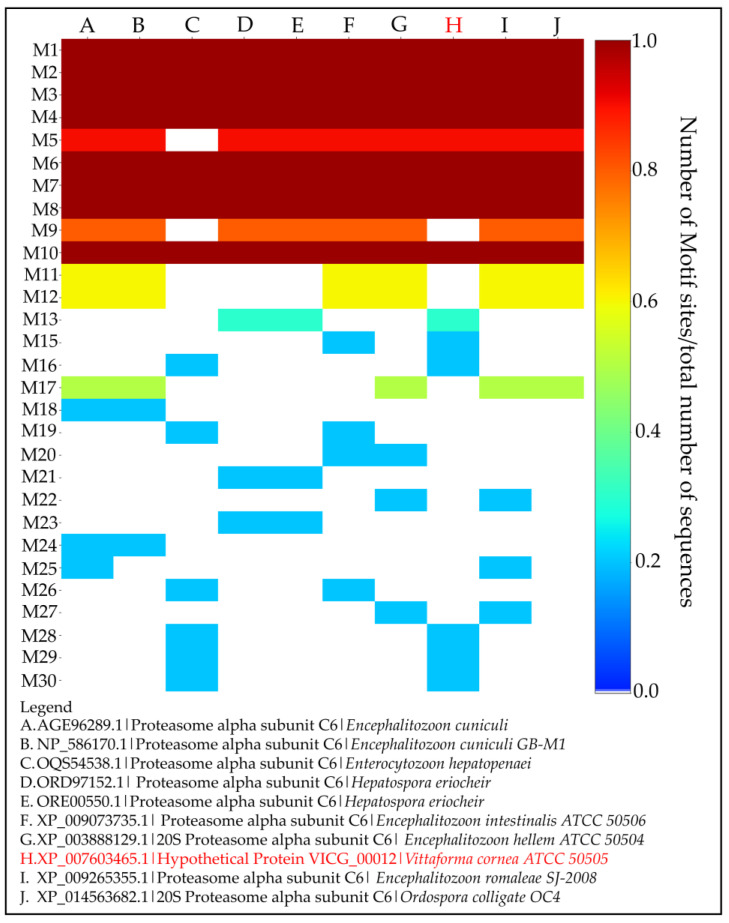
The motif analysis assessment of VICG00012 predicted it as a proteasome alpha subunit protein (PSA), highlighted in red. The motif analysis heatmap generated from MEME Suite highlighting 6 highly conserved motifs across the PSA homologs listed in the legend (A–J) shows the similarity among these samples. Motif 9 was missing in the HP and proteasome alpha subunit in *Enterocytozoon hepatopenaei*, while motifs 16 and 28–30 were present in these two species.

A total of eight motifs were highly conserved across all the 10 homologous sequences retrieved from BLASTp results obtained from PHASE I of the methodology pipeline (Figure 5). Moreover, the HP lacked motif 9, which was conserved across other species except for *Encephalitozoon hepatopenaei*, a species phylogenetically classified in the same group as *Vittaforma corneae* [87]. A close-up analysis of the conserved motifs in all species and those conserved in a few sets of sequences are further analysed at the residue and structural level (see Sections c and d below, respectively).

*c.*  *Multiple Sequence Alignment and Motif Mapping*

**Figure 6:**
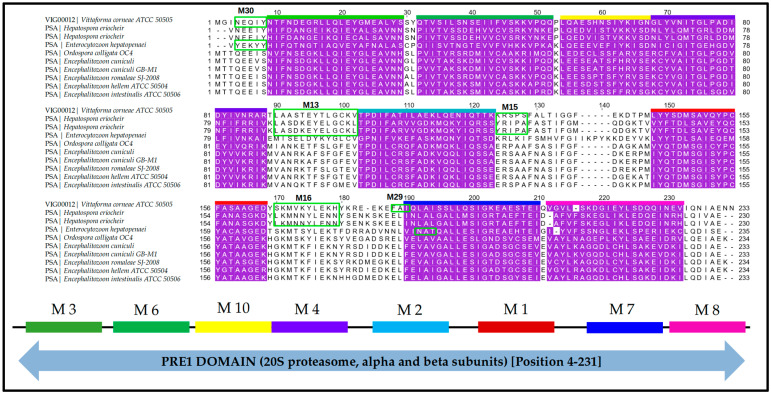
The motifs identified by MEME mapped onto the multiple sequence alignment results of the HP VICG00012 and its homologs. Multiple sequencing alignment was performed using MUSCLE [88]. The motif numbering is based on the MEME results. Motifs conserved in all sequences are highlighted in purple, while those found in only a subset of species within the same group are represented in green boxes. Motif identities are represented in different-coloured bars and labelled accordingly. The motifs 13, 15, and 16 were conserved in *V. corneae* and *H. eriocheir*, while motifs 28–30 were conserved in *V. corneae* and *E. hepatopenaei*. The conserved domain identified in this sequence belonged to the PRE1 superfamily spanning across the entire sequence, as shown in the diagrammatic representation below the alignment.

Sequence analysis showed high similarity across the selected set of proteins. Mapping the motifs to the multiple sequence alignments showed slight differences at the residue level (Figure 6). This was consistent with the separate clades identified further in phylogenetic analysis where species in the same group had similar residues within the conserved motifs but were different from the other groups. Regular expressions of the sequence patterns from these motifs are shown in Table 1. Motifs 13, 15, 16, and 28–30 were present in VICG00012 and several varying microsporidia species while missing in others (Figure 6). Motif 13 (regular expression: LA[SA][DS][KT]EY[ET]LGC) was observed in VICG00012 from *V. corneae* (motif sequence: LAASTEYTLGCKV) and *H. eriocheir* (motif sequence: LASDKEYELGCKL). Motif 15 (regular expression: RS) was similarly observed in both the HP (motif sequence: KRS) and *E. intestinalis* (motif sequence: YRS) with a change in residue K and Y. Moreover, motif 16 (regular expression: YLEK[HT][FY][DK]) was observed in VICG00012 (motif sequence: YLEKHYK) and *H. eriocheir* (motif sequence: YLENNYS). Motifs 28–30 (regular expressions: [FM]G; [FN]A[IT]; [NY]E[KQ][IY], respectively) were observed only in *V. corneae* and *E. hepatopenaei*. Proteins within this subset were subsequently shown to cluster together within the same clade after tree calculations (see Section f below).

*d.*  *Motif Mapping to Homology Models*

Homology modelling of VICG00012 was performed using the *Leishmania tarentolae* proteasome 20S subunit protein (PDB ID: 6QM7) template. Target-template alignment was performed using T-COFFEE. Predicted models with the lowest z-DOPE score were selected and their structural quality was checked using Verify3D [89], quantitative model energy analysis (QMEAN) [90], PROCHECK [91], and protein structure analysis (ProSA-web) [92] (Table S6). The Verify3D tool was used to verify the accuracy of the predicted protein models with its one-dimensional amino acid sequence [89], and showed that 86.61% of the residues in the modelled HP had a score greater or equal to 0.2. The QMEAN, on the other hand, is a quality assessment tool from the SWISS-MODEL server that incorporates several structural measurements with a resultant z-score [90]. In this particular model, the QMEAN z-score was −3.84. The ProSA web server checks for potential errors in 3D models displayed in a plot with z-score deviations from the baseline mean considered erroneous [92]. The ProSA z-score of the 3D structure was −7.59, while the PROCHECK validation test further illustrated that a total of 90.6% of residues were located in the most favoured regions in the model. All these tests collectively validated the accuracy of the predicted structure. 

**Figure 7:**
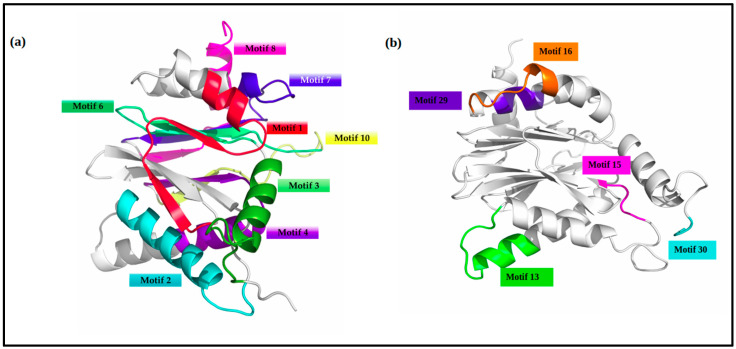
Motif mapping to the homology model of the hypothetical protein VICG00012 is highlighted in two planes of view. Motifs numbers are based on MEME outputs. (**a**) A total of 8 highly conserved motifs were identified. The motif colours here are consistent with the formatting in the alignment mapping shown previously. (**b**) The second plane of view of the HP highlights the unique motifs 13, 15, 16, and 29. These motifs were found in *Vittaforma corneae* and its close relative *Enterocytozoon hepatopenaei*.

A 3D model of the VICG00012 is highlighted in Figure 7. The eight conserved motifs identified among all species were mapped onto the predicted structure using PyMOL (Figure 7). Motifs 13, 15, 16, and 29 were conserved in *V. corneae* and the *Enterocytozoon hepatopenaei’s* protein Proteasome Alpha subunit C6. Motifs 2–4, 13, and 29 were characterized with alpha-helix coils while motifs 1, 6, 7, 8, and 10 mapped onto beta sheets in the predicted 3D model. The range of the start and end positions of each motif is highlighted in Table 2.

*e.*  *Identification and Analysis of Key Domains*

The HP VICG00012 contains the N-terminal of the proteasome alpha subunit (Figure 6) PRE1 domain, spanning from position 4–231. The PRE1 domain is a primary component of the 20S proteasome structure and has been shown to be actively involved in post-translational modification, chaperones, and protein turnover [93,94,95].

*f.*  *Phylogenetic Tree Calculations and Pairwise Sequence Identity Calculations*

**Figure 8:**
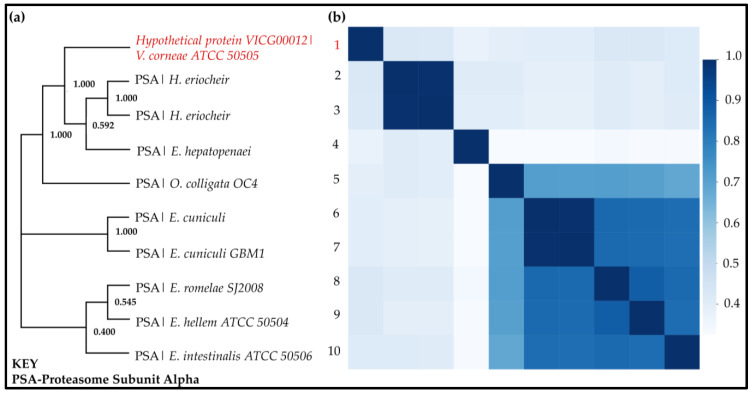
Phylogenetic classification of HP VICG00012 (UniProt ID: L2GQV7) is shown in red. (**a**) Tree calculation was performed on MEGA X. Phylogeny construction was performed using the maximum likelihood heuristic search method and the Le–Gascuel 2008 model (+I, parameter = 0.11) at 90% site coverage (1000 bootstrap replicates). The initial tree was generated using the default neighbour-joining/BioNJ algorithm. The best tree with the highest log-likelihood of −2737.423 is shown. (**b**) Sequence identity heatmap highlighting a pairwise comparison of each of the sequences used in the phylogenetic analysis. A darker colour shows a closer identity to respective sequences. Two distinct clusters are observed with higher identity in the Encephalitozoon family compared to the clade containing the HP of interest.

Tree analysis of the proteasome subunit alpha sequences homologous to VICG00012 identified three separate clusters (Figure 8a). The HP VICG00012 was more closely related to the *Hepatospora eriocheir, Enterocytozoon hepatopenaei*, and *Ordospora colligata OC4* species which infect crustaceans, while the other clades in the evolutionary tree infect mammals [87]. This was also observed with the regular expressions of the motifs identified in these microorganisms in Table 1 above and as discussed in Section c. This similarity further suggests that this may be a fast-evolving gene among microsporidia that aids survival within different infection localization patterns. 

Pairwise sequence identity was concordant with the tree analysis groupings showing higher sequence identities among the separated clades (Figure 8b). Moreover, the second and third clades had a higher sequence similarity than the first group containing the HP. This high similarity could be attributed to the fact that the species in the last two groups all infect mammalian hosts [87,96]. 

#### 2.3.2. Case Study II: Functional and Structural Analysis of VICG01314

The similarity search identified the HP VICG01314 (Accession number: XP_007604760.1) as the ribosomal protein L3 which plays an active role in protein translation and is localized in the ribosome. It forms part of the large ribosomal subunit. STRING protein association prediction highlighted this protein to be in close proximity to other regulatory and enzymatic proteins with varying functions such as translation initiation factor, synthases, kinases, and GTP-binding proteins that would assist in the regulation of the metabolic process in which the HP would most likely play a role (Figure S1). 

This HP consists of 18 well-conserved motifs within the 10 homologous sequences (Figure S2). Motif 20, which is nine residues long, was present in all sequences apart from the HP. Multiple sequence alignment and motif mapping highlight very few residue-level differences within these sequences (Figure S3 and Supplementary File S2). The HP contains the ribosomal protein L3 domain spanning from residue 1 to 358 out of a total length of 383 amino acids. All conserved motifs were found within this domain. Additionally, motif 23 (PGMKY[ET]DL) was conserved in only *V. corneae* and *E. hepatopenaei*, while motifs 27 (Y[EK][DI) and 29 (GF[ST) were conserved in *V. corneae* and *H. eriocheir* (Supplementary File S2). All three of these microsporidia were subsequently found to be grouped phylogenetically.

Phylogenetic analysis and pairwise sequence identity calculations determined that the sequences were of close evolutionary distances (Figure S4). However, in this case, phylogeny shows that the HP VICG01314 clustered away from the rest of the clades but was closer relative to *H. eriocheir* and *E. hepatopenaei* (Figure S4a). This was a similar trend to what was observed with the HP VICG00012 reported above. Furthermore, this was evident with the two distinct clusters in the heatmap, where a slightly darker cluster is observed among VICG01314, *H. eriocheir*, and *E. hepatopenaei* (Figure S4b). The other seven sequences form the second cluster, with a higher pairwise identity in the group containing *E. hellem, E. romelae*, and *E. intestinalis*—parasites infecting mammalian hosts. The 3D model of VICG01314 was structured using the 6AZ3 chain B template, a ribosomal protein L3 from the Leishmania parasite (Figure S5). The most distinct motifs were also mapped onto this structure. 

#### 2.3.3. Case Study III: Functional and Structural Analysis of VICG01349

The HP VICG01349 (Accession number: XP_007604795.1) is classified as the E2F-dimerization-partner transcription factor of the dimerization domain of DP (DP-DD) superfamily. The dimerization partner binds to E2F transcription factors, forming heterodimers that plan integral functions in regulating genes involved in DNA synthesis, apoptosis, cell cycle progression, and proliferation [97]. Its key interacting protein based on STRING prediction includes similar proteins in the cyclin family involved in cell-cycle regulation and DNA polymerase and other synthesis proteins involved in a similar role (Figure S6).

This protein is predicted to be localized in the cell nucleus and contains a signal peptide and a noncytoplasmic domain (Figure S7). The protein sequence contains a DNA-binding winged-helix motif on the N-terminal and a DP domain on the C-terminal. Moreover, motif analysis identified that this protein and its homologs have a total of seven conserved motifs (Figure S7). There were no unique motifs absent or present solely in the HP. However, it is important to note that five motifs were only present in *V. corneae* and *Enterospora canceri*, alluding to the close similarity between these two protein sequences. These were motifs 16 (F[IM]), 17 (R[ES]), 23 ([LS][FG]), 25 (D[AK]KK[HL]), and 30 (LR[EL]). Multiple sequence alignment and motif mapping according to numbers provided by MEME Suite output are shown in Figure S8, highlighting this relationship. 

The phylogenetic evaluation determined that these sequences were less divergent (Figure S9a). The protein showed higher identity with both mammalian-specific and crustacean-specific microsporidia that were grouped; *E. bieneusi* (affecting mammals), *E. canceri*, and *H. eriocheir* (the last two affecting crustaceans) (Figure S9b) [87], and thus improved confidence in the inference of the protein function of the HP from information available from its closest relatives. Homology modelling of the HP was performed using the protein transferase from *Oryctolagus cuniculus* (PDB ID: 1CF7) and the *Homo sapiens* E2F4-DP1 homolog (PDB ID: 5TUU). Highly conserved motifs were then mapped onto the structure (Figure S10).

#### 2.3.4. Case Study IV: Functional and Structural Analysis of VICG01687

Helicases are enzymes that bind to double-stranded DNA, separating them into single-stranded DNA during DNA replication. VICG01687 belongs to the superfamily I DNA-RNA Helicase (Accession number: XP_007605132.1). The STRING association network analysis showed close interaction with other binding proteins including the nuclear cap-binding protein, GTP-binding, ribosomal L18e/L15P, hydrolases, and the DEAD-box helicase proteins (Figure S11). These neighbouring proteins work in conjunction with the HP, based on their proximity and classification within the same superfamily.

Additionally, these proteins contain a P-loop motif. Twenty-two highly conserved motifs were detected across homologous sequences (Figure S12), illustrating that the sequence similarity within this group was quite high and that the conserved motifs would play an important function in the protein’s functioning. 

The HP L2GL01 is categorized as a member of superfamily 1 C-terminal helicase domain under the Upf1-like helicases family (SF1_C_Upf1) and contains the DEAD domain involved in ATP-dependent RNA and DNA winding [82] (Figure S13). Its conserved features include an ATP-binding site and a DNA-binding site and function in transcription regulation [98].

Phylogenetic analysis identified three distinct clusters (Figure S14a). The VICG01687 also clustered together with microorganisms infecting invertebrates and distinctly separate from the microsporidia of mammals. Pairwise sequence identity calculations and the generated heatmap also highlighted this relationship (Figure S14b). The predicted 3D structure was modelled using the human zinc-finger hydrolase protein (PDB ID: 2WJY) known to regulate nonsense transcripts. Motif mapping is highlighted in Supplementary Figure S15. 

#### 2.3.5. Case Study V: Functional and Structural Analysis of VICG01723

The HP VICG01723 (Accession number: XP_007605168.1) was classified as ATP-Dependent RNA Helicase (SKI2 RNA Helicase) based on the homology search. Association networks place this protein in close relationship with other uncharacterized proteins, poly(A) polymerase, the Nop-domain-containing protein, and the WD-repeats region domain-containing protein (Figure S16). The Nop domain is found in pre-RNA ribonucleoproteins and contains RNA- and protein-binding surfaces [98].

The conserved domain search grouped this HP as a Superfamily II RNA helicase involved in RNA replication, recombination, and repair. The sequence contains three distinct domains: the DEAD-box domain which plays various roles in RNA metabolism, the conserved helicase carboxyl-terminal domain often found in conjunction with the DEAD domain, and the DSHCT domain [82,98]. In addition, this protein was particularly distinctive based on the very large number of conserved motifs identified. Twenty-eight out of thirty conserved motifs were reported by MEME analysis (Figure S17). Moreover, Motifs 28 and 27 appeared to be missing from VICG01723, with the former present in all other species. Multiple sequence alignment and motif mapping show the positions of some of these motifs along the sequence (Figure S18). 

In agreement with the previous annotations, evolutionary relationships indicated that the HP clusters in the same clade as other characterized RNA helicase proteins from microsporidia infecting invertebrates, that is, *Nosema ceranae* and *Nosema granulosis* (Figure S19a). Moreover, the tree highlights the divergence of this HP away from similar proteins isolated from microsporidian species affecting the same host, an observation also made by the high sequence identity among the latter group (Figure S19b). This shows the versatility of the evolution of the fast-adapting microsporidia species and could probably be an effect of horizontal gene transfer or gene loss over time that has led to this divergence [14,19,99].

The modelling of the VICG01723 protein was performed using four templates that spanned across the length of the protein, providing better coverage compared to using a single template with poor coverage. The templates used included the antiviral RNA helicase SKI2 protein from *Saccharomyces cerevisiae S288C* (PDB ID: 4BUJ and 5MC6), *Saccharomyces cerevisiae* (PDB ID: 4A4Z), human RNA helicase (PDB ID: 6IEH), and *Neurospora crassa* (PDB ID: 6BB8). The identified motifs were similarly mapped onto the 3D structure (Figure S20).

## 3. Materials and Methods

As a first step prior to this study, an extensive literature search was performed on *Vittaforma corneae* proteins on Google Scholar [100] and Scopus [101] using the key search terms “*Vittaforma corneae* hypothetical proteins”; “*Vittaforma corneae* unknown proteins characterization”; and “Function and structure prediction of *Vittaforma corneae* unknown proteins”. This search revealed no relevant research on this specific microorganism. Subsequently, the study was designed, and the pipeline followed for the annotation of the HPs is illustrated in Figure 1. This is divided into two main phases: PHASE I consists of the identification and retrieval of unique HPs from online databases and homology search; PHASE II highlights the key tools used in the functional annotation of the selected proteins including protein family classification, characterization of the physicochemical properties of the HPs, prediction of membrane proteins and presence of signal peptides, identification of their interacting proteins, and the metabolic pathways. Table S1 highlights the main tools and online resources used for the characterization of these proteins.

### 3.1. Sequence Retrieval

The proteome of *Vittaforma corneae ATCC 50505 (Nosema corneum)* was retrieved from the Microsporidia Database in FASTA format [102] (data retrieved on 8 December 2022). A total of 2034 (90.97%) of the 2237 proteins were identified as hypothetical, and a custom Python script was used to filter these sequences. The code worked by identifying any sequence headers that contained the term “HYPOTHETICAL” and stored these headers and their respective sequences in a separate FASTA file. To ensure that all retrieved sequences were not repeated, the code also checked the sequences against each other, and unique sequences were retained. From this clean-up, a total of 164 HPs were included in the final subset used in the following analyses. 

### 3.2. Sequence Similarity Search

A remote BLAST+ command-line sequence search of the 2034 HPs against the NCBI BLASTp standard nonredundant protein sequences (NR) database was performed [32,103] (database accessed in December 2022). This crucial step aimed to identify homologous protein sequences with known functions. This search was conducted using default parameters including an E-value of 0.001 and a bit-score greater than or equal to 200, limiting the search to a maximum number of target sequences of 10. After the first search, 627 protein sequences were revealed to have no similar sequences in the database, while 677 sequences had homologous sequences in the NCBI database. The Position-Specific Iterative Basic Local Alignment Search Tool (PSI-BLAST) was additionally used to iteratively search for distantly related sequences by using position-specific matrices (PSSMs) to score matches between the query and database [104] (database accessed between 8–16 December 2022). Notably, PSI-BLAST is comparatively similar to the HMMER (jackhmmer) program’s working algorithm [58]. The scores of the 627 sequences lacking homologs in BLASTp and PSI-BLAST indicated either no similar sequences, similarity to HPs from other microsporidians, or poor sequence similarity and coverage to any hits on the database (Supplementary File S1). These were therefore excluded from the study since the focus was on inferring function from information available on other known proteins from other microsporidia species.

Moreover, to identify true homology and filter out similar sequences that might have resulted from chance, another more stringent BLAST search was performed on the homologous hits of the HPs using an in-house Python script. Python v. 3.8 was used to generate the code [105,106,107,108]. The code functioned by running command line BLAST+ and filtered the BLAST output hits where the HP appeared in the top three with high coverage (>50%), percent identity (>40%), and e-value (<0.005). These were considered true homologs of the respective HP. A schematic presentation of the reverse BLAST technique to identify true homology is highlighted in the Supplementary Materials (Figure S21). These were then used for the following downstream in silico characterization processes (*n* = 164). 

### 3.3. Classification of Protein Families

Several freely available online resources were used to identify protein families, and these included Pfam v34. [33] and InterPro [34] tools embedded in the HMMer web server [109]. The top hit with the most statistically significant search output was used to categorize the protein families (significance E-values for hits = 0.03 and sequence = 0.01). Additional tools used in identifying protein families included PANTHER [35], PRINTS [36], PIRSF [37], and GENE3D [38]. 

### 3.4. Subcellular Localization

Protein function is often linked to the location of the protein within the cell. For example, most signalling and transport proteins are found localized within the membrane [77]. To predict the localization of the HPs included in this study, various online tools were used, including TMHMM v. 2.0 [47,77,110], DeepTMHMM [111], TargetP-2.0 [45], SignalP-5.0 [46,112], and Phobius [48] (data accessed on 12 January 2023). TMHMM uses the hidden Markov model to predict the presence of transmembrane helices in a protein [47,77,110]. Additionally, TargetP, Phobius, and SignalP were used to predict the presence of signal peptides and the location of cleavage sites within the protein. 

### 3.5. Protein Characterization by Physicochemical Properties

The ExPASy-web server’s ProtParam tool computes several physical and chemical properties of the HPs [44] (data accessed on 12 January 2023). The output was set to determine the properties of each protein including molecular weight (MW), theoretical isoelectric point (pI), amino acid composition, atomic composition, extinction coefficient, instability index, aliphatic index, estimated half-life, and the grand average of hydropathicity (GRAVY). Batch processing of the physicochemical properties was performed using an in-house Jupyter Lab v. 1.2.6 script that ran a remote command-line search of the HPs against the ExPASy program using default settings that allowed the entire sequence to be analysed.

### 3.6. Protein Interaction Network Analysis

STRING version 11 was used to predict protein interaction networks [49,50]. STRING is a computational protein interaction prediction database that uses information from other related organisms and from the known interactions stored in other primary databases providing either physical or functional associations between proteins. Identification of HP interaction using this database was performed by selecting the organism *Vittaforma corneae ATCC 50505* and searching the uncharacterized protein by sequence using default parameters (full STRING network type and medium confidence score of 0.400). The networks assessed in this study were based on interaction evidence from laboratory experiments.

### 3.7. Phylogenetic Tree Calculations and Pairwise Sequence Identity

Multiple sequence alignment (MSA) was performed on the set of HPs whose functional annotation was confirmed, and sequence similarity search was significantly based on the E-value. Alignment was performed using the Multiple Sequence Comparison by Log-Expectation (MUSCLE) algorithm [88] in the Molecular Evolutionary Genetics Analysis (MEGA version X) software [113]. Batch phylogenetic tree calculations were also conducted in MEGA version X, using the command line MEGA-CC route [114]. First, the best three maximum likelihood tree models were identified for each set of HPs and were used to set runs on the command line. For each set of aligned proteins, nine runs were conducted, each producing two outputs: the consensus and test neighbour-joining trees at 1000 Bootstrap replicates. The generated trees were compared for similarity with the consensus trees. Trees that did not match with the consensus were considered “failed” runs, and realignment was considered. Tree visualization was performed using Dendroscope version 3.7.4 [115]. The five best trees with bootstrap values greater than 50% were used for subsequent annotations as case studies. These were VICG00012, VICG01314, VICG01349, VICG01687, and VICG01723. Moreover, an all versus all pairwise sequence identity calculation for each protein sequence set was performed using an in-house Python script and a heatmap generated in Jupyter Notebook v. 1.2, as described in [116]. 

### 3.8. Functional Annotation-Functional Domains Prediction and Structural Analysis

Identification of protein superfamilies and conserved functional domains was performed using the online NCBI Batch CD-Search (data retrieved and re-evaluated in December 2022) maintaining the default search mode, that is, against the CDD v. 3.19 tool [39,40] at a set E-value cut-off of 10^−4^ with filtered low-complexity regions and composition-corrected scoring. CDD annotates proteins by identifying their domain architecture and, hence, predicting protein structure, function, superfamily classification, and relationships. The search was limited to specific hits and all nonspecific hits were disregarded.

Identification of motifs within the sets of similar proteins was performed using the Multiple Expectation-maximization for Motif Elicitation (MEME) version 5.3.3 [117] and Motif Alignment and Search Tool (MAST) version 5.3.3 [118], tools included in the motif discovery MEME Suite online platform [43]. The MEME command used sought a maximum number of 15 motifs in the sequences with motif lengths ranging from 3–20 residues [119], a range of 2–10 sites per motif, and an E-value of less than 0.05. MAST was used to analyse the significance of these motifs identified using *p*-values by sorting the sequences in descending order starting with the best match to all motifs with an E-value of less than 10 and a position *p*-value of less than 10^−5^. The MAST output identifies the least significant motifs that need to be filtered out from the MEME output. Where redundant motifs were identified, an MAST rerun was conducted, removing these motifs. Jalview v. 2.11.1.4 [120] was further used to map motifs onto the respective multiple sequence alignments. Conservation heatmaps were generated from each key motif identified from the MEME output log files using a Python script [116]. This was performed by calculating the total number of sites per total number of subgroup sequences [119]. 

The EXPASY ScanProsite tool was used to identify functional and structurally important intradomain residues [121] against the UniProt Knowledgebase (UniProtKB) [122] SwissProt database. The search was restricted to the Fungi group.

### 3.9. Gene Ontology and Metabolic Pathway Analysis

The Kyoto Encyclopedia of Genes and Genomes (KEGG) is an online open-source database that provides information on the key metabolic pathways in which a protein is involved [123]. The database contains a range of categories. This tool identifies the metabolic processes involved between the microbe and its host. KEGG version 104.00 (data accessed on 5 January 2023) was used to identify main pathways using the KofamKOALA (KEGG Orthology And Links Annotation) analysis tool which assigns K-numbers to sequences using HMMSEARCH against the customized KEGG Orthologs HMM database. Significant scores are highlighted in the output with an asterisk (Table S5). 

### 3.10. Homology Modelling

Template identification was performed using PRIMO [51] and HHPred [124]. The best template was selected based on the percent sequence identity, high coverage, and high resolution (2–3.80 Å). The templates used in this study are highlighted in Table 3 below. Moreover, pairwise secondary structure alignment of the query and template were analysed to assess the quality of the alignment and confirm that the selected template was the best for subsequent homology modelling. One hundred models were calculated by MODELLER v. 9.23 [124] using the very slow refinement mode. The top three models with the lowest z-DOPE score were selected for validation. The best models were selected based on quality assessments using ProSA [92], PROCHECK [91], QMEAN [125], and VERIFY3D [89]. PyMOL [126] was used to map highly conserved motifs onto the 3D model (Table S6). Only motifs within the plane of view were labelled on each predicted structure. Motif numbering was based on output labels from the motif-discovery MEME Suite runs. 

## 4. Conclusions

Various bioinformatics approaches are used in the characterization of unknown proteins and the discovery of their primary biochemical and physical properties. With the improvement in the efficiency of tools for the in silico identification of important proteins that may be further investigated via laboratory experimentation, the processing time of unknown proteins has substantially improved. Several studies highlight significant findings on the functions of HPs from microorganisms that are useful in deciphering their biology and modes of infection. This study highlights the use of such a pipeline in annotating the functions and structures of some HPs from the microsporidian parasite, *Vittaforma corneae ATCC 50505 (Nosema corneae)*. Similar to most microsporidian species, a large section of the HPs in this study were identified to be involved in regulatory and transport functions, which is key for the survival of these unique microorganisms due to their compact genomes and extreme levels of gene loss. Altogether, this study also identifies several conserved regions in the HP and their relatives within the same clade that could also be included in the ever-growing set of conserved proteins used in the taxonomical classification of the fast-evolving microsporidia species. This paper further compares different online tools in classifying their respective families and function, with a clear consistency of results across different platforms. It proceeds further to describe steps associated with modelling the 3D structures of several case study genes, serving as a demonstration of the capacity of computational methods to discern the structure and function of the unknown from the known. From this investigation, it is apparent that accurate and proper annotation of HPs is possible and valuable in deciphering new structures, functions, and pathways. A similar annotation pipeline can therefore be used for the initial characterization of new proteins from newly sequenced genomes and provide a better understanding of the biology of the microorganism of interest. Nevertheless, it is important to note that the computational approach of protein annotation used in this study is just the initial step in elucidating the HPs’ function. Therefore, *in vivo*, and *in vitro* experimental validation steps are still needed to further confirm these inferred functions. Admittedly, this report solely predicts the 3D structures of HPs but falls short of determining their importance in the infection biology of the microsporidia as a whole. However, this structural information is pivotal for future studies that could use these models to perform an extensive evaluation of the HPs’ interactions with host proteins and to impact drug discovery and diagnostics.

## Figures and Tables

**Table 1:** Regular expression patterns from identified motifs in HP VICG00012 and homologous proteins from other microsporidia species.

Motif #	Regular Expression	Occurrence
Motif 1	[ML]S[GA][IV][SE]Y[PQ]C[YF]A[TN]A[AS]G[EK][KD][HY][GLS]K	Present in all species
Motif 2	TPDI[LF][CA]R[SV][FV][AG]DK[MIV]Q[KQ][LY]IQ[SRT]	Present in all species
Motif 3	[NH]IF[ND][SA][DNE]G[KE][LI][LK]QIEY[GA]L[ES]A[VL]	Present in all species
Motif 4	[KN][CL]Y[VL][AGQ][IM]TG[LR][PL][GD]D[IVMT][DN][YF][IV][VFI][KNR]R[IA	Present in all species
Motif 5	AF[NA][AS][ST]IFG[FM][DQ][KDG][EG][KR][PAT][MV][IV]Y[QF]T	Absent only in *E. hepatopenaei*
Motif 6	[PQ][IV][VI][TS][VA][KS]S[KDR][DSE][MEH][IV]V[CF][VA]S[KR]K[IVY][PN][QKR	Present in all species
Motif 7	ALL[EM]SIG[ART][DET][SA][GE][CF][ST]E[IV][ED][VA][AFG][YV][LF	Present in all species
Motif 8	[QE][DG]L[CEI][YHK]L[SE][AD][KQE]E[IV][DN][KR][IHV]LQ[DI][IV]A	Present in all species
Motif 9	[KN][FY][IL]E[KN][NS]Y[RS][ES][DN][IKM][DES][DK][KE]EL[FI][EN][LV]	Absent in *V. corneae* and *E. hepatopenaei*
Motif 10	[DE][KP]L[EQ]E[SED][ESV][AIS][ST][ST][FIV][YHK][KR][VI]S[ED	Present in all species
Motif 11	KMVANRK[TA]FS[FL]	Absent in *V. corneae*
Motif 12	MTTQEE[IV	Absent in *V. corneae*
Motif 13	LA[SA][DS][KT]EY[ET]LGC	Present only in *V. corneae* and *H. eriocheir*
Motif 15	RS	Present in *V. corneae* and *E. intestinalis*
Motif 16	YLEK[HT][FY][DK]	Present only in *V. corneae* and *E. hepatopenaei*
Motif 17	[AE]ER[SP]	Absent in *V. corneae*
Motif 18	NG	Absent in *V. corneae*
Motif 19	M[EH]	Absent in *V. corneae*
Motif 20	RA	Absent in *V. corneae*
Motif 21	NEEI	Absent in *V. corneae*
Motif 22	NA	Absent in *V. corneae*
Motif 23	NS	Absent in *V. corneae*
Motif 24	FE	Absent in *V. corneae*
Motif 25	KA	Absent in *V. corneae*
Motif 26	N[NS]	Absent in *V. corneae*
Motif 27	TE	Absent in *V. corneae*
Motif 28	[FM]G	Present only in *V. corneae* and *E. hepatopenaei*
Motif 29	[FN]A[IT]	Present only in *V. corneae* and *E. hepatopenaei*
Motif 30	[NY]E[KQ][IY]	Present only in *V. corneae* and *E. hepatopenaei*

**Table 2:** Start and end positions of motifs identified among the HP VICG00012 and its homologs from different species.

Sample ID/Motif #	M1	M2	M3	M4	M6	M7	M8	M10	M13	M15	M16	M28	M29	M30
*VICG0012*	148–168	103–123	9–29	68–88	32–52	190–210	212–231	52–68	90–102	125–128	170–178	1–4	182–186	4–9
*Encephalitozoon cuniculi*	148–168	103–123	9–29	68–88	32–52	191–211	214–234	52–68	-	-	-	-	-	-
*Encephalitozoon cuniculi GB-M1*	148–168	103–123	9–29	68–88	32–52	191–211	214–234	52–68	-	-	-	-	-	-
*Enterocytozoon hepatopenaei*	151–171	101–121	7–27	66–86	30–50	194–214	216–236	50–66	-	-	173–181	132–135	190–194	2–7
*Hepatospora eriocheir*	146–166	101–121	7–27	66–86	30–50	189–209	211–231	50–66	88–100	-	-	-	-	-
*Hepatospora eriocheir*	146–166	101–121	7–27	66–86	30–50	189–209	211–231	50–66	88–100	-	-	-	-	-
*Encephalitozoon intestinalis ATCC 50506*	148–168	103–123	9–29	68–88	32–52	191–211	214–234	52–68	-	125–128	-	-	-	-
*Encephalitozoon hellem ATCC 50504*	148–168	103–123	9–29	68–88	32–52	191–211	214–234	52–68	-	-	-	-	-	-
*Encephalitozoon romaleae SJ-2008*	148–168	103–123	9–29	68–88	32–52	191–211	214–234	52–68	-	-	-	-	-	-
*Ordospora colligata OC4*	148–168	103–123	9–29	68–88	32–52	191–211	214–234	52–68	-	-	-	-	-	-

“-” represents missing motifs in respective cells.

**Table 3:** A summary of the selected templates used to model each HP.

HP ID	Template PDB ID	Sequence Identity (%)	Coverage (%)	Resolution (Å)
VICG00012	6QM7	25	98	2.80
VICG01314	6AZ3	60	98	2.50
VICG01349	1CF7	19	35	2.60
	5TUU	24	41	2.25
VICG01687	2WJY_A	43	98	2.50
VICG01723	4BUJ	36	93	3.70
	5MC6	38	93	3.80
	4A4Z	38	93	2.40
	6IEH	44	98	2.89
	6BB8	45	94	3.49

## Data Availability

All the data are presented in this article and the Supplementary Materials. Publicly available datasets were analysed in this study.

## Supplementary Materials

The following supporting information can be downloaded at: https://www.mdpi.com/article/10.3390/ijms24043507/s1.
