# Supplementary material for: Structural and Functional Annotation of Hypothetical Proteins from the Microsporidia Species Vittaforma corneae ATCC 50505 Using in silico Approaches
Source: Int J Mol Sci. 2023 Feb 9;24(4):3507. doi: 10.3390/ijms24043507 (PMC9960886; doi:10.3390/ijms24043507)
Supplement: Supplementary file 1 [file ijms-24-03507-s001.zip › ijms-1953007-supplementary.pdf]

Article

Structural and Functional Annotation of Hypothetical Proteins from the Microsporidia Species *Vittaforma corneae ATCC 50505* Using In silico Approaches

Supplementary Materials

Lilian Mbaisi Ang'ang'o [1], Jeremy Keith Herren [2] and Özlem Tastan Bishop [1,*]

[1] Research Unit in Bioinformatics (RUBi), Department of Biochemistry and Microbiology, Rhodes University, Makhanda 6140, South Africa
[2] International Centre of Insect Physiology and Ecology, Nairobi P.O. Box 30772-00100, Kenya
* Correspondence: o.tastanbishop@ru.ac.za

Table S1. A list of bioinformatic tools and resources used for the functional annotation of *Vittaforma corneae ATCC 50505* Hypothetical Proteins (HP).

Annotation Type	Software	URL	Reference
Sequence retrieval and similarity search	NCBI-BLAST	https://blast.ncbi.nlm.nih.gov/Blast.cgi	[1]
	MicrosporidiaDB	https://microsporidiadb.org/	[2]
	UniProt	https://www.uniprot.org/	[3]
Programming languages for script generation	Python v. 3.8	https://www.python.org/	[4]
	Jupyter Notebook	https://jupyter.org/	[5]
Protein family classification	Pfam	http://pfam.xfam.org	[6]
	InterPro	https://www.ebi.ac.uk/interpro/	[7]
	HMMer	https://www.ebi.ac.uk/Tools/hmmer/	[8]
	PANTHER	http://www.pantherdb.org	[9]
	PRINTS	http://130.88.97.239/PRINTS/index.php	[10]
	PIRSF	https://proteininformationresource.org/pirsf/	[11]
Sequence alignment and phylogenetic analysis	MEGA X	https://www.megasoftware.net/	[12,13]
	MUSCLE	NA	[14]
	Dendroscope v. 3.7.4	https://uni-tuebingen.de/en/fakultaeten/mathematisch-naturwissenschaftliche-fakultaet/fachbereiche/informatik/lehrstuehle/algorithms-in-bioinformatics/software/dendroscope/	[15]
Motif analysis and domain functional annotation	MEME Suite (MEME & MAST)	https://meme-suite.org	[16]
	EXPASY-ScanProsite	https://prosite.expasy.org/scanprosite/	[17]
	CDD Search	https://www.ncbi.nlm.nih.gov/Structure/bwrpsb/bwrpsb.cgi	[18]
	SMART	https://smart.embl.de	[19]
Characterization of physicochemical properties	EXPASY-PROTPARAM	https://web.expasy.org/protparam/	[20]
Subcellular localization	TMHMM v. 2.0	http://www.cbs.dtu.dk/services/TMHMM/	[21]
	TargetP v. 2.0	http://www.cbs.dtu.dk/services/TargetP/	[22]
	SignalP v. 5.0	http://www.cbs.dtu.dk/services/SignalP/	[23]
	Phobius	https://www.ebi.ac.uk/Tools/pfa/phobius/	[24]
Protein-protein association network	STRING v. 11 (Search tool for the retrieval of interacting genes and proteins)	https://string-db.org/	[25]
Gene Ontology	KEGG	https://www.genome.jp/kegg/	[26]
Homology Modeling	PRIMO	https://primo-oligo.rubi.ru.ac.za/	[27]
	MODELLER	https://salilab.org/modeller/	[28]

PROSA-web	https://prosa.services.came.sbg.ac.at/prosa.php	[29]
VERIFY3D	https://saves.mbi.ucla.edu/	[30]
PROCHECK	https://www.ebi.ac.uk/thornton-srv/software/PROCHECK/	[31]
QMEAN	http://swissmodel.expasy.org/qmean	[32]
SWISS-MODEL	https://swissmodel.expasy.org	[33]

Table S2. A summary of the functional categories of the annotated HPs.

**Predicted Function**		**HPs (NCBI Accession numbers)**
1. Enzymatic Activity	Hydrolase	XP_007603465.1, XP_007603484.1, XP_007603568.1, XP_007603726.1, XP_007603893.1, XP_007604190.1, XP_007604316.1, XP_007604934.1, XP_007605007.1, XP_007605048.1, XP_007605091.1, XP_007605128.1, XP_007605132.1, XP_007605168.1, XP_007605243.1, XP_007605249.1, XP_007605468.1
	Phosphatase	XP_007605069.1, XP_007605421.1
	Kinase	XP_007603954.1, XP_007604292.1
	Transferase	XP_007603549.1, XP_007603706.1, XP_007604045.1, XP_007604313.1, XP_007604512.1, XP_007604572.1, XP_007604786.1, XP_007605348.1, XP_007605513.1
	Redoxin	XP_007603620.1, XP_007604320.1
	Reductase	XP_007604630.1
2. Binding Proteins	DNA Binding	XP_007604222.1, XP_007603555.1, XP_007605148.1, XP_007603976.1, XP_007604702.1, XP_007604711.1, XP_007605014.1, XP_007605563.1, XP_007604217.1, XP_007605600.1, XP_007604795.1, XP_007604991.1, XP_007605236.1
	RNA Binding	XP_007603502.1, XP_007603756.1, XP_007604025.1, XP_007603684.1, XP_007604059.1, XP_007603503.1, XP_007605101.1
	Protein Binding	XP_007603813.1, XP_007604439.1, XP_007604066.1, XP_007604530.1, XP_007604889.1, XP_007605221.1, XP_007605241.1, XP_007605535.1, XP_007604255.1, XP_007604458.1, XP_007604339.1,
	ATP Binding	XP_007605468.1, XP_007605243.1, XP_007603484.1, XP_007604934.1, XP_007605249.1, XP_007605007.1, XP_007605091.1, XP_007604805.1, XP_007605346.1, XP_007603899.1, XP_007604183.1, XP_007604190.1, XP_007604236.1, XP_007605168.1
	Metal ion Binding	XP_007603620.1, XP_007604027.1, XP_007605126.1, XP_007605437.1, XP_007603868.1, XP_007604818.1, XP_007604533.1, XP_007603568.1, XP_007603571.1, XP_007603586.1, XP_007603651.1, XP_007604813.1, XP_007605092.1
	Lipid Binding	XP_007603764.1

3. Other Proteins	Transport	XP_007605235.1, XP_007605292.1, XP_007603481.1, XP_007605504.1, XP_007603688.1, XP_007603592.1, XP_007603628.1, XP_007605468.1
	Structural	XP_007604846.1, XP_007605182.1, XP_007605382.1, XP_007605476.1, XP_007603578.1, XP_007604054.1, XP_007604133.1, XP_007604178.1, XP_007604216.1, XP_007604260.1, XP_007604760.1, XP_007604961.1, XP_007605096.1, XP_007605153.1, XP_007605458.1
	Cellular/ Regulatory	XP_007603642.1, XP_007604801.1, XP_007604849.1, XP_007603534.1, XP_007604051.1, XP_007603959.1, XP_007604109.1, XP_007604802.1, XP_007603650.1, XP_007604335.1, XP_007604344.1, XP_007603773.1, XP_007603728.1
	Cell-cycle Related Proteins	XP_007604773.1, XP_007604450.1, XP_007604487.1, XP_007604653.1, XP_007603902.1, XP_007603465.1, XP_007603726.1, XP_007603972.1, XP_007604824.1

Table S3. List of annotated HP functional groups.

NCBI Accession No.	UniProt ID	NCBI Sequence ID	Proteins/Domains and functions
XP_007603465.1	L2GQV7	hypothetical protein VICG_00012	Proteasome Subunit Alpha/Beta
XP_007603481.1	L2GPA0	hypothetical protein VICG_00028	Importin N-Terminal Domain-Containing Protein
XP_007603484.1	L2GPG0	hypothetical protein VICG_00031	ABC Transporter Domain-Containing Protein
XP_007603502.1	L2GQ49	hypothetical protein VICG_00049	60S Ribosome Subunit Biogenesis Protein NIP7
XP_007603503.1	L2GQF3	hypothetical protein VICG_00050	Ribosomal_L18e/L15P Domain-Containing Protein
XP_007603524.1	L2GPJ6	hypothetical protein VICG_00071	Uncharacterized Protein
XP_007603534.1	L2GPK6	hypothetical protein VICG_00081	Protein MAK16
XP_007603539.1	L2GPL1	hypothetical protein VICG_00086	Uncharacterized Protein
XP_007603546.1	L2GPG2	hypothetical protein VICG_00093	Rab-GAP TBC Domain-Containing Protein
XP_007603549.1	L2GPL9	hypothetical protein VICG_00096	SAM_MT_RSMB_NOP Domain-Containing Protein
XP_007603554.1	L2GPM3	hypothetical protein VICG_00101	Uncharacterized Protein
XP_007603555.1	L2GR40	hypothetical protein VICG_00102	DNA Polymerase Epsilon Catalytic Subunit, EC 2.7.7.7
XP_007603565.1	L2GR49	hypothetical protein VICG_00112	Uncharacterized Protein
XP_007603568.1	L2GQK5	hypothetical protein VICG_00115	UPF0613 PROTEIN PB24D3.06C/ Alpha/Beta-Hydrolases
XP_007603571.1	L2GPI5	hypothetical protein VICG_00118	Cytosolic Iron-Sulfur Assembly Component 2b
XP_007603578.1	L2GQL3	hypothetical protein VICG_00125	F-Box And Leucine-Rich Repeat Protein 17
XP_007603586.1	L2GPK0	hypothetical protein VICG_00133 (partial sequence)	Regulatory Protein Mig1-Related
XP_007603592.1	L2GQC9	hypothetical protein VICG_00139	Major Facilitator Superfamily Domain-Containing Protein 1
XP_007603620.1	L2GR97	hypothetical protein VICG_00167	2Fe-2S Ferredoxin-Type Domain-Containing Protein
XP_007603628.1	L2GQR6	hypothetical protein VICG_00175	ATP-Binding Cassette Sub-Family B Member 7, Mitochondrial
XP_007603642.1	L2GQH2	hypothetical protein VICG_00189	BRCT Domain-Containing Protein
XP_007603650.1	L2GRC7	hypothetical protein VICG_00197	HTH TFE/IIealpha-Type Domain-Containing Protein
XP_007603651.1	L2GPQ9	hypothetical protein VICG_00198	Uncharacterized Protein
XP_007603657.1	L2GQI3	hypothetical protein VICG_00204	Uncharacterized Protein
XP_007603684.1	L2GPZ9	hypothetical protein VICG_00231	Pre-RRNA-Processing Protein PNO1
XP_007603688.1	L2GQX8	hypothetical protein VICG_00235	Protein Transport Protein SEC23
XP_007603698.1	L2GQZ1	hypothetical protein VICG_00245	ANK_REP_REGION Domain-Containing Protein
XP_007603706.1	L2GPW4	hypothetical protein VICG_00253 (partial sequence)	SET Domain-Containing Protein
XP_007603726.1	L2GNV6	hypothetical protein VICG_00273	Proteasome Subunit Alpha Type-2
XP_007603728.1	L2GNQ5	hypothetical protein VICG_00275	SBDS Domain-Containing Protein
XP_007603756.1	L2GNY8	hypothetical protein VICG_00303	Brix Domain-Containing Protein
XP_007603764.1	L2GPM6	hypothetical protein VICG_00311	Tricalbin-1-Related
XP_007603773.1	L2GNV7	hypothetical protein VICG_00320	SAS-6_N Domain-Containing Protein
XP_007603795.1	L2GQ38	hypothetical protein VICG_00342	RNA-Directed RNA Polymerase, EC 2.7.7.48

XP_007603813.1	L2GP17	hypothetical protein VICG_00360	PFU Domain-Containing Protein
XP_007603861.1	L2GPA6	hypothetical protein VICG_00408	Uncharacterized Protein
XP_007603868.1	L2GP55	hypothetical protein VICG_00415	RING-Type Domain-Containing Protein
XP_007603872.1	L2GQS9	hypothetical protein VICG_00419	Gpp34
XP_007603878.1	L2GP60	hypothetical protein VICG_00425	USP Domain-Containing Protein
XP_007603893.1	L2GPU4	hypothetical protein VICG_00440	Ruvb-Like Helicase, EC 3.6.4.12
XP_007603899.1	L2GN71	hypothetical protein VICG_00446	T-Complex Protein 1 Subunit Zeta
XP_007603902.1	L2GND6	hypothetical protein VICG_00449	Ubiquitin-Like Domain-Containing Protein
XP_007603942.1	L2GNH6	hypothetical protein VICG_00490	GTP--RNA Guanylyltransferase (EC 2.7.7.50) (MRNA Guanylyltransferase)
XP_007603954.1	L2GNC7	hypothetical protein VICG_00502	ATP-Dependent (S)-NAD(P)H-Hydrate Dehydratase (EC 4.2.1.93) (ATP-Dependent NAD(P)HX Dehydratase)
XP_007603959.1	L2GND2	hypothetical protein VICG_00507	GINS Helical Bundle-Like
XP_007603972.1	L2GNK7	hypothetical protein VICG_00520	Swi5
XP_007603976.1	L2GPI8	hypothetical protein VICG_00524	Histone Domain-Containing Protein
XP_007603978.1	L2GQ23	hypothetical protein VICG_00526	Ero1-Related
XP_007603994.1	L2GNH1	hypothetical protein VICG_00542	VPS9 Domain-Containing Protein
XP_007603998.1	L2GQ42	hypothetical protein VICG_00546	MPN Domain-Containing Protein
XP_007604004.1	L2GNI2	hypothetical protein VICG_00552	Uncharacterized Protein
XP_007604025.1	L2GPC3	hypothetical protein VICG_00573	KRR-R Motif-Containing Protein 1
XP_007604027.1	L2GNQ7	hypothetical protein VICG_00575	B Box-Type Domain-Containing Protein
XP_007604045.1	L2GPE8	hypothetical protein VICG_00593	Phosphatidate Cytidylyltransferase (EC 2.7.7.41)
XP_007604046.1	L2GPR1	hypothetical protein VICG_00594	Uncharacterized Protein
XP_007604051.1	L2GPR6	hypothetical protein VICG_00599	SM Domain-Containing Protein
XP_007604054.1	L2GNN0	hypothetical protein VICG_00602	Actin
XP_007604058.1	L2GQA4	hypothetical protein VICG_00606	Uncharacterized Protein
XP_007604059.1	L2GNN6	hypothetical protein VICG_00607	PUA Domain-Containing Protein
XP_007604066.1	L2GPT1	hypothetical protein VICG_00614	Leucine Rich Repeat Containing Protein
XP_007604109.1	L2GMY6	hypothetical protein VICG_00657	CBFD_NFYB_HMF Domain-Containing Protein
XP_007604133.1	L2GPP5	hypothetical protein VICG_00682	40S Ribosomal Protein S14/30s Ribosomal Protein S11
XP_007604137.1	L2GN78	hypothetical protein VICG_00686	Uncharacterized Protein
XP_007604178.1	L2GPT0	hypothetical protein VICG_00727	MICSWaP
XP_007604183.1	L2GPT3	hypothetical protein VICG_00732	Heat Shock Cognate 70
XP_007604190.1	L2GNY7	hypothetical protein VICG_00739 (partial sequence)	Chromodomain-Helicase-DNA-Binding Protein 3-Related-Related
XP_007604198.1	L2GMR6	hypothetical protein VICG_00747	Eukaryotic Translation Initiation Factor 2c
XP_007604216.1	L2GNE8	hypothetical protein VICG_00765	40s Ribosomal Protein S24
XP_007604217.1	L2GNS0	hypothetical protein VICG_00766	Meiotic Nuclear Division Protein 1
XP_007604222.1	L2GNS4	hypothetical protein VICG_00771	DNA Polymerase Alpha Subunit B
XP_007604236.1	L2GNG7	hypothetical protein VICG_00787	TP6A_N Domain-Containing Protein
XP_007604246.1	L2GNH4	hypothetical protein VICG_00797	U-Box Domain-Containing Protein
XP_007604255.1	L2GMP6	hypothetical protein VICG_00806	WD_REPEATS_REGION Domain-Containing Protein

XP_007604260.1	L2GMQ0	hypothetical protein VICG_00811	60s Ribosomal Protein L32 And DNA-Directed RNA Polymerase II, Subunit N
XP_007604265.1	L2GMQ4	hypothetical protein VICG_00816	Uncharacterized Protein
XP_007604292.1	L2GNX8	hypothetical protein VICG_00843	Tnase-Like Domain-Containing Protein
XP_007604313.1	L2GNX0	hypothetical protein VICG_00864	Histone Acetyltransferase (EC 2.3.1.48)
XP_007604316.1	L2GNE5	hypothetical protein VICG_00867	AAA Domain-Containing Protein
XP_007604320.1	L2GN26	hypothetical protein VICG_00871	Glutaredoxin Domain-Containing Protein
XP_007604335.1	L2GN44	hypothetical protein VICG_00886	NOT2_3_5 Domain-Containing Protein
XP_007604339.1	L2GMC1	hypothetical protein VICG_00890	Pbs Lyase Heat-Like Protein
XP_007604344.1	L2GMC8	hypothetical protein VICG_00897	Ribosome Biogenesis Regulatory Protein
XP_007604350.1	L2GN62	hypothetical protein VICG_00903	Xaa-Pro Aminopeptidase 1
XP_007604410.1	L2GM75	hypothetical protein VICG_00963	CMP/Dcmp-Type Deaminase Domain-Containing Protein
XP_007604439.1	L2GN97	hypothetical protein VICG_00992	TPR_REGION Domain-Containing Protein
XP_007604450.1	L2GMB5	hypothetical protein VICG_01003	Kinetochore Protein SPC25
XP_007604458.1	L2GMZ1	hypothetical protein VICG_01011	WD_REPEATS_REGION Domain-Containing Protein
XP_007604487.1	L2GNF9	hypothetical protein VICG_01041	NOC3p Domain-Containing Protein
XP_007604512.1	L2GNI6	hypothetical protein VICG_01066	Glycine Hydroxymethyltransferase (EC 2.1.2.1) (Serine Methylase)
XP_007604530.1	L2GN20	hypothetical protein VICG_01084	WD40 Repeat Protein Swd2

Table S4. Subcellular localization prediction of HPs using different online tools. The hydrophobic proteins (highlighted in pink) also had membrane-spanning regions.

UNIPROT Accession Number	Sequence ID	GRAVY SCORE	Grand Average of Hydropathy (GRAVY)	Membrane prediction using TargetP	Membrane prediction using SignalP	Membrane prediction using DeepTMHMM	Membrane region prediction using TMHMM	TMHMM: Y/N indicator if a transmembrane region was predicted or not	Phobius: Y/N indicator if a signal peptide was predicted or not (SP)
L2GIT5	VICG_02177	Hydrophilic	-0.69676	OTHER	OTHER	GLOB	Topology=o	NULL	NULL
L2GIY9	VICG_02113	Hydrophilic	-0.11802	OTHER	OTHER	GLOB	Topology=o	NULL	Y
L2GJC9	VICG_02001	Hydrophilic	-0.70339	OTHER	OTHER	GLOB	Topology=i	NULL	NULL
L2GJD5	VICG_02006	Hydrophilic	-0.88433	OTHER	OTHER	GLOB	Topology=o	NULL	NULL
L2GJF9	VICG_01972	Hydrophilic	-0.39593	OTHER	OTHER	GLOB	Topology=i	NULL	NULL
L2GJH0	VICG_01905	Hydrophilic	-0.87866	OTHER	OTHER	GLOB	Topology=o	NULL	NULL
L2GJH1	VICG_02179	Hydrophobic	0.082759	OTHER	OTHER	GLOB	Topology=o	NULL	NULL
L2GJH3	VICG_01970	Hydrophobic	0.688824	OTHER	OTHER	TM	Topology=i7-29o44-66i87-109o122-144i	Y	NULL
L2GJH7	VICG_02174	Hydrophobic	0.114655	OTHER	OTHER	GLOB	Topology=o	NULL	NULL
L2GJK3	VICG_01935	Hydrophilic	-0.32743	OTHER	OTHER	SP	Topology=o	NULL	Y
L2GJK7	VICG_01923	Hydrophobic	0.061765	SP	SP(Sec/SPI)	SP+TM	Topology=o106-128i	Y	Y
L2GJM0	VICG_02133	Hydrophilic	-0.25259	SP	OTHER	SP	Topology=i12-31o	Y	NULL
L2GJM1	VICG_01861	Hydrophilic	-1.06019	OTHER	OTHER	GLOB	Topology=o	NULL	NULL
L2GJM4	VICG_01938	Hydrophilic	-1.06094	OTHER	OTHER	GLOB	Topology=o	NULL	NULL
L2GJN1	VICG_02124	Hydrophilic	-0.04245	OTHER	OTHER	GLOB	Topology=o	NULL	NULL
L2GJP3	VICG_01869	Hydrophilic	-0.81527	OTHER	OTHER	GLOB	Topology=o	NULL	NULL
L2GJU3	VICG_01837	Hydrophilic	-0.14605	OTHER	OTHER	GLOB	Topology=o	NULL	NULL
L2GJX6	VICG_01777	Hydrophilic	-1.00604	OTHER	OTHER	GLOB	Topology=o	NULL	NULL
L2GK39	VICG_01700	Hydrophilic	-0.14393	OTHER	OTHER	GLOB	Topology=o	NULL	NULL
L2GK50	VICG_01710	Hydrophobic	0.674902	OTHER	OTHER	TM	Topology=i50-72o82-101i122-144o164-186i199-221o	Y	NULL

L2GK83	VICG_01745	Hydrophobic	0.240476	OTHER	OTHER	GLOB	Topology=o213-235i	Y	NULL
L2GKB3	VICG_01980	Hydrophilic	-0.54874	OTHER	OTHER	GLOB	Topology=o	NULL	NULL
L2GKD6	VICG_02005	Hydrophilic	-0.69649	OTHER	OTHER	GLOB	Topology=o	NULL	NULL
L2GKD7	VICG_01689	Hydrophilic	-0.95541	OTHER	OTHER	GLOB	Topology=o	NULL	NULL
L2GKF9	VICG_01887	Hydrophobic	0.566839	OTHER	OTHER	TM	Topology=i13-35o50-72i79-101o170-192i	Y	NULL
L2GKH5	VICG_01597	Hydrophilic	-0.21944	SP	OTHER	SP	Topology=i7-29o	Y	NULL
L2GKK6	VICG_02135	Hydrophilic	-0.45833	OTHER	OTHER	GLOB	Topology=o	NULL	NULL
L2GKK9	VICG_01848	Hydrophilic	-0.30722	OTHER	OTHER	GLOB	Topology=o	NULL	NULL
L2GKL3	VICG_01927	Hydrophilic	-0.12158	OTHER	OTHER	GLOB	Topology=o	NULL	Y
L2GKM8	VICG_01768	Hydrophilic	-0.59106	SP	SP(Sec/SPI)	SP	Topology=o	NULL	Y
L2GKQ8	VICG_01808	Hydrophobic	0.481992	OTHER	OTHER	TM	Topology=o104-126i135-157o181-203i230-252o	Y	NULL
L2GKR9	VICG_01556	Hydrophobic	0.065373	SP	SP(Sec/SPI)	SP	Topology=o	NULL	Y
L2GKU1	VICG_01738	Hydrophobic	0.648669	OTHER	OTHER	SP+TM	Topology=o63-85i104-126o136-155i172-194o209-231i	Y	Y
L2GKU8	VICG_01465	Hydrophilic	-0.79352	OTHER	OTHER	GLOB	Topology=o	NULL	NULL
L2GKY1	VICG_01987	Hydrophilic	-1.05979	OTHER	OTHER	GLOB	Topology=o	NULL	NULL
L2GKZ1	VICG_01677	Hydrophilic	-0.24596	OTHER	OTHER	GLOB	Topology=o	NULL	NULL
L2GKZ7	VICG_01754	Hydrophilic	-0.55	OTHER	OTHER	TM	Topology=i13-35o	Y	Y
L2GL19	VICG_01951	Hydrophilic	-0.16023	OTHER	OTHER	GLOB	Topology=o	NULL	NULL
L2GL34	VICG_01799	Hydrophilic	-0.24781	OTHER	OTHER	GLOB	Topology=o	NULL	NULL
L2GL53	VICG_01914	Hydrophilic	-0.74837	OTHER	OTHER	GLOB	Topology=o	NULL	NULL
L2GL85	VICG_01509	Hydrophilic	-0.61302	OTHER	OTHER	GLOB	Topology=o	NULL	NULL
L2GLA1	VICG_01658	Hydrophilic	-0.73554	OTHER	OTHER	GLOB	Topology=o	NULL	NULL
L2GLC5	VICG_01274	Hydrophilic	-0.99966	OTHER	OTHER	GLOB	Topology=o	NULL	NULL
L2GLD5	VICG_01693	Hydrophobic	0.515054	OTHER	OTHER	TM	Topology=o40-62i83-105o159-181i	Y	NULL
L2GLD6	VICG_01289	Hydrophilic	-0.27742	OTHER	OTHER	GLOB	Topology=o	NULL	NULL
L2GLE9	VICG_01212	Hydrophilic	-0.35167	OTHER	OTHER	GLOB	Topology=o	NULL	NULL
L2GLF0	VICG_01304	Hydrophilic	-0.02918	OTHER	OTHER	TM	Topology=i17-39o	Y	NULL

L2GLF8	VICG_01900	Hydrophilic	-0.07978	OTHER	OTHER	GLOB	Topology=o15-33i	Y	NULL
L2GLG3	VICG_01319	Hydrophilic	-0.26066	OTHER	OTHER	GLOB	Topology=o	NULL	NULL
L2GLG9	VICG_01816	Hydrophobic	0.583077	OTHER	OTHER	TM	Topology=i65-87o107-129i	Y	NULL
L2GLH2	VICG_01200	Hydrophobic	0.20625	OTHER	OTHER	GLOB	Topology=i21-42o	Y	NULL
L2GLH3	VICG_01237	Hydrophilic	-0.62159	OTHER	OTHER	GLOB	Topology=o	NULL	NULL
L2GLH9	VICG_01826	Hydrophilic	-0.711	OTHER	OTHER	GLOB	Topology=o	NULL	NULL
L2GLI9	VICG_01611	Hydrophilic	-0.13504	OTHER	OTHER	GLOB	Topology=o	NULL	NULL
L2GLK5	VICG_01756	Hydrophilic	-0.96766	OTHER	OTHER	TM	Topology=o11-30i	Y	Y
L2GLN1	VICG_01408	Hydrophobic	0.502076	OTHER	OTHER	TM	Topology=i100-122o132-151i186-208o223-245i258-280o	Y	NULL
L2GLR3	VICG_01180	Hydrophobic	0.458904	OTHER	OTHER	TM	Topology=i12-34o49-71i92-114o147-169i	Y	NULL
L2GLS7	VICG_01153	Hydrophilic	-0.68	OTHER	OTHER	GLOB	Topology=o	NULL	NULL
L2GLT7	VICG_01741	Hydrophobic	0.539643	OTHER	OTHER	SP+TM	Topology=i12-34o72-94i115-137o152-171i184-206o226-248i	Y	NULL
L2GLZ3	VICG_01384	Hydrophilic	-0.93077	OTHER	OTHER	GLOB	Topology=i	NULL	NULL
L2GM02	VICG_01567	Hydrophilic	-0.05888	OTHER	OTHER	GLOB	Topology=o	NULL	NULL
L2GM06	VICG_01282	Hydrophilic	-0.10743	OTHER	OTHER	GLOB	Topology=o	NULL	NULL
L2GM09	VICG_01399	Hydrophilic	-0.30317	OTHER	OTHER	GLOB	Topology=o	NULL	Y
L2GM27	VICG_01307	Hydrophilic	-0.73058	OTHER	OTHER	GLOB	Topology=o	NULL	NULL
L2GM39	VICG_01322	Hydrophilic	-0.49864	OTHER	OTHER	GLOB	Topology=o	NULL	NULL
L2GM40	VICG_00975	Hydrophilic	-0.08995	OTHER	OTHER	SP	Topology=o	NULL	Y
L2GM46	VICG_01613	Hydrophilic	-0.188	OTHER	OTHER	GLOB	Topology=o	NULL	NULL
L2GM50	VICG_00985	Hydrophilic	-0.09758	OTHER	OTHER	GLOB	Topology=o	NULL	NULL
L2GM69	VICG_01502	Hydrophilic	-0.37422	OTHER	OTHER	SP	Topology=o	NULL	NULL
L2GM73	VICG_01507	Hydrophilic	-1.01338	SP	SP(Sec/SPI)	SP	Topology=o	NULL	Y
L2GM79	VICG_01243	Hydrophilic	-0.25938	OTHER	OTHER	GLOB	Topology=o	NULL	NULL
L2GM83	VICG_01248	Hydrophobic	0.082308	SP	SP(Sec/SPI)	GLOB	Topology=o	NULL	Y
L2GM89	VICG_01030	Hydrophilic	-0.51852	OTHER	OTHER	GLOB	Topology=o	NULL	NULL
L2GMA7	VICG_00875	Hydrophilic	-0.49032	OTHER	OTHER	GLOB	Topology=o	NULL	NULL

L2GMB3	VICG_01363	Hydrophilic	-0.44333	OTHER	OTHER	GLOB	Topology=o	NULL	NULL
L2GMB6	VICG_00885	Hydrophilic	-0.6404	OTHER	OTHER	GLOB	Topology=o	NULL	NULL
L2GMF4	VICG_00922	Hydrophilic	-0.93396	OTHER	OTHER	GLOB	Topology=i	NULL	NULL
L2GMF7	VICG_01461	Hydrophilic	-0.31196	OTHER	OTHER	SP	Topology=o	NULL	Y
L2GMG7	VICG_01186	Hydrophilic	-0.63333	OTHER	OTHER	GLOB	Topology=o	NULL	NULL
L2GMH3	VICG_01481	Hydrophilic	-0.27984	OTHER	OTHER	GLOB	Topology=o	NULL	NULL
L2GMH8	VICG_01323	Hydrophilic	-0.29854	OTHER	OTHER	TM	Topology=o166-188i	Y	NULL
L2GMI3	VICG_00937	Hydrophilic	-0.263	OTHER	OTHER	TM	Topology=o67-89i	Y	NULL
L2GMI7	VICG_01204	Hydrophilic	-0.40321	OTHER	OTHER	GLOB	Topology=o	NULL	NULL
L2GMK7	VICG_01229	Hydrophobic	0.178713	OTHER	OTHER	GLOB	Topology=o	NULL	NULL
L2GML3	VICG_00764	Hydrophobic	0.042508	OTHER	OTHER	GLOB	Topology=o	NULL	NULL
L2GML4	VICG_00925	Hydrophilic	-0.19878	OTHER	OTHER	GLOB	Topology=o	NULL	NULL
L2GMS2	VICG_00956	Hydrophilic	-0.95016	OTHER	OTHER	GLOB	Topology=o	NULL	NULL
L2GMT4	VICG_00767	Hydrophilic	-0.04833	OTHER	OTHER	TM	Topology=i21-40o	Y	NULL
L2GMW2	VICG_01197	Hydrophilic	-0.03108	SP	SP(Sec/SPI)	GLOB	Topology=o	NULL	Y
L2GN29	VICG_00630	Hydrophilic	-0.5927	OTHER	OTHER	GLOB	Topology=o	NULL	NULL
L2GN32	VICG_00876	Hydrophilic	-0.81125	OTHER	OTHER	GLOB	Topology=i	NULL	NULL
L2GN65	VICG_00671	Hydrophobic	0.57619	OTHER	OTHER	GLOB	Topology=o	NULL	NULL
L2GN78	VICG_00686	Hydrophobic	0.373671	OTHER	OTHER	GLOB	Topology=o10-32i63-85o95-117i136-158o173-195i	Y	NULL
L2GN81	VICG_00456	Hydrophobic	0.673799	OTHER	OTHER	TM	Topology=i84-106o121-143i150-172o	Y	NULL
L2GN87	VICG_00461	Hydrophilic	-0.97283	OTHER	OTHER	GLOB	Topology=o	NULL	NULL
L2GN88	VICG_00696	Hydrophilic	-0.43852	SP	SP(Sec/SPI)	SP	Topology=o10-27i	Y	Y
L2GN89	VICG_00982	Hydrophilic	-0.86299	OTHER	OTHER	GLOB	Topology=o	NULL	NULL
L2GN93	VICG_00987	Hydrophilic	-0.42963	OTHER	OTHER	GLOB	Topology=o	NULL	NULL
L2GN99	VICG_00711	Hydrophilic	-0.63924	OTHER	OTHER	GLOB	Topology=o	NULL	NULL
L2GNA9	VICG_01154	Hydrophilic	-0.9	OTHER	OTHER	GLOB	Topology=o	NULL	NULL
L2GNB2	VICG_00726	Hydrophilic	-0.44824	OTHER	OTHER	GLOB	Topology=o	NULL	NULL

L2GNB7	VICG_00492	Hydrophilic	-0.659	OTHER	OTHER	GLOB	Topology=o	NULL	NULL
L2GNC3	VICG_01017	Hydrophilic	-1.02007	OTHER	OTHER	GLOB	Topology=o	NULL	NULL
L2GNG8	VICG_00887	Hydrophilic	-0.8613	OTHER	OTHER	GLOB	Topology=o	NULL	NULL
L2GNJ0	VICG_01071	Hydrophilic	-0.55788	OTHER	OTHER	GLOB	Topology=o	NULL	NULL
L2GNJ1	VICG_00505	Hydrophilic	-0.52288	OTHER	OTHER	GLOB	Topology=i	NULL	NULL
L2GNK4	VICG_00832	Hydrophilic	-0.14173	OTHER	OTHER	GLOB	Topology=o	NULL	NULL
L2GNK8	VICG_00924	Hydrophilic	-0.28768	OTHER	OTHER	GLOB	Topology=o	NULL	NULL
L2GNN2	VICG_00550	Hydrophilic	-0.68673	OTHER	OTHER	GLOB	Topology=o	NULL	NULL
L2GNQ2	VICG_00969	Hydrophilic	-0.3242	OTHER	OTHER	GLOB	Topology=o	NULL	NULL
L2GNR8	VICG_00984	Hydrophilic	-0.50437	OTHER	OTHER	GLOB	Topology=o	NULL	NULL
L2GNS8	VICG_00674	Hydrophobic	0.036254	SP	SP(Sec/SPI)	SP	Topology=o	NULL	Y
L2GNT1	VICG_00783	Hydrophilic	-0.46058	OTHER	OTHER	GLOB	Topology=o	NULL	NULL
L2GNU4	VICG_00798	Hydrophilic	-0.17024	OTHER	OTHER	GLOB	Topology=o	NULL	NULL
L2GNX6	VICG_00724	Hydrophilic	-0.63729	OTHER	OTHER	GLOB	Topology=o	NULL	NULL
L2GNY0	VICG_00874	Hydrophilic	-1.02951	OTHER	OTHER	GLOB	Topology=o	NULL	NULL
L2GP13	VICG_00328	Hydrophilic	-0.51106	OTHER	OTHER	GLOB	Topology=o	NULL	NULL
L2GP35	VICG_00385	Hydrophilic	-0.54934	SP	SP(Sec/SPI)	GLOB	Topology=o	NULL	Y
L2GP51	VICG_00410	Hydrophobic	0.90708	OTHER	OTHER	TM	Topology=o31-53i66-88o117-139i159-181o196-218i	Y	NULL
L2GP54	VICG_00513	Hydrophilic	-1.11364	OTHER	OTHER	GLOB	Topology=o	NULL	NULL
L2GP81	VICG_00008	Hydrophilic	-0.25846	OTHER	OTHER	GLOB	Topology=o	NULL	NULL
L2GP87	VICG_00730	Hydrophilic	-0.27687	OTHER	OTHER	TM	Topology=i81-103o118-140i	Y	NULL
L2GPA4	VICG_00558	Hydrophilic	-0.09459	OTHER	OTHER	GLOB	Topology=o	NULL	NULL
L2GPB0	VICG_00563	Hydrophilic	-0.81575	OTHER	OTHER	GLOB	Topology=o	NULL	NULL
L2GPC0	VICG_00790	Hydrophobic	0.565789	OTHER	OTHER	TM	Topology=i9-31o41-61i82-104o119-141i154-176o	Y	NULL
L2GPF8	VICG_00830	Hydrophilic	-0.58931	OTHER	OTHER	GLOB	Topology=o	NULL	NULL
L2GPG1	VICG_00603	Hydrophilic	-0.36027	OTHER	OTHER	GLOB	Topology=o	NULL	NULL
L2GPH0	VICG_00103	Hydrophilic	-0.24812	OTHER	OTHER	GLOB	Topology=o	NULL	NULL

L2GPH3	VICG_00845	Hydrophilic	-0.88365	OTHER	OTHER	TM	Topology=o	NULL	NULL
L2GPJ3	VICG_00529	Hydrophilic	-0.12813	OTHER	OTHER	GLOB	Topology=o	NULL	NULL
L2GPJ9	VICG_00631	Hydrophobic	0.364205	OTHER	OTHER	GLOB	Topology=o36-58i118-140o155-177i189-211o	Y	NULL
L2GPM2	VICG_00656	Hydrophobic	0.017411	OTHER	OTHER	GLOB	Topology=o	NULL	NULL
L2GPM3	VICG_00101	Hydrophilic	-0.00758	OTHER	OTHER	GLOB	Topology=o	NULL	NULL
L2GPN2	VICG_00316	Hydrophilic	-0.99076	OTHER	OTHER	GLOB	Topology=o	NULL	NULL
L2GPN5	VICG_00672	Hydrophilic	-0.105	OTHER	OTHER	GLOB	Topology=i	NULL	NULL
L2GPP1	VICG_00574	Hydrophilic	-0.87179	OTHER	OTHER	GLOB	Topology=o	NULL	NULL
L2GPP3	VICG_00326	Hydrophilic	-0.82799	OTHER	OTHER	GLOB	Topology=o	NULL	NULL
L2GPQ4	VICG_00136	Hydrophilic	-0.725	OTHER	OTHER	GLOB	Topology=o	NULL	NULL
L2GPQ8	VICG_00702	Hydrophilic	-0.93121	OTHER	OTHER	GLOB	Topology=o	NULL	NULL
L2GPT2	VICG_00223	Hydrophilic	-0.76561	OTHER	OTHER	GLOB	Topology=o	NULL	NULL
L2GPV0	VICG_00366	Hydrophilic	-0.57225	OTHER	OTHER	GLOB	Topology=o	NULL	NULL
L2GPV1	VICG_00282	Hydrophobic	0.015966	OTHER	OTHER	SP	Topology=i20-42o	Y	NULL
L2GPW9	VICG_00297	Hydrophilic	-0.74894	OTHER	OTHER	GLOB	Topology=o	NULL	NULL
L2GPX0	VICG_00201	Hydrophilic	-0.96047	OTHER	OTHER	GLOB	Topology=o	NULL	NULL
L2GPX1	VICG_00391	Hydrophilic	-0.26957	OTHER	OTHER	GLOB	Topology=o	NULL	NULL
L2GPX8	VICG_00268	Hydrophobic	0.070879	OTHER	OTHER	GLOB	Topology=o	NULL	NULL
L2GQ03	VICG_00327	Hydrophilic	-0.99679	OTHER	OTHER	GLOB	Topology=o	NULL	NULL
L2GQ24	VICG_00019	Hydrophilic	-0.36092	SP	SP(Sec/SPI)	SP	Topology=o	NULL	Y
L2GQ27	VICG_00024	Hydrophilic	-0.07576	OTHER	OTHER	GLOB	Topology=o	NULL	NULL
L2GQ45	VICG_00044	Hydrophilic	-0.58009	OTHER	OTHER	GLOB	Topology=o	NULL	NULL
L2GQ71	VICG_00074	Hydrophilic	-0.88452	OTHER	OTHER	GLOB	Topology=o	NULL	NULL
L2GQ78	VICG_00387	Hydrophilic	-0.44378	OTHER	OTHER	GLOB	Topology=o	NULL	NULL
L2GQD1	VICG_00020	Hydrophilic	-0.43085	SP	SP(Sec/SPI)	SP	Topology=o	NULL	Y
L2GQH1	VICG_00329	Hydrophilic	-0.49825	OTHER	OTHER	GLOB	Topology=o	NULL	NULL
L2GQJ0	VICG_00100	Hydrophilic	-0.17608	OTHER	OTHER	GLOB	Topology=o	NULL	NULL

L2GQM7	VICG_00364	Hydrophilic	-1.0297	OTHER	OTHER	GLOB	Topology=o	NULL	NULL
L2GQQ6	VICG_00165	Hydrophilic	-0.35819	OTHER	OTHER	TM	Topology=i288-310o314-336i	Y	NULL
L2GQT9	VICG_00429	Hydrophilic	-0.11149	SP	OTHER	SP	Topology=o	NULL	Y
L2GQW9	VICG_00225	Hydrophilic	-1.235	OTHER	OTHER	GLOB	Topology=o	NULL	NULL
L2GQZ4	VICG_00052	Hydrophilic	-0.92065	OTHER	OTHER	GLOB	Topology=i	NULL	NULL
L2GQZ8	VICG_00057	Hydrophilic	-0.81976	OTHER	OTHER	GLOB	Topology=o	NULL	NULL
L2GR92	VICG_00162	Hydrophilic	-0.69771	OTHER	OTHER	GLOB	Topology=o	NULL	NULL
L2GRB3	VICG_00182	Hydrophobic	0.144531	SP	SP(Sec/SPI)	SP+TM	Topology=o93-115i	Y	Y
L2GRJ2	VICG_00267	Hydrophilic	-1.06728	OTHER	OTHER	GLOB	Topology=o	NULL	NULL

Table S5: A summary of the proteins involved in KEGG Gene Ontology and metabolic pathways.

# **Gene name**	**KO**	**threshold**	**score**	**E-value**	**KO definition**
* XP_007603502.1hypotheticalproteinVICG_00049	K07565	39.47	195.2	9.70E-60	60S ribosome subunit biogenesis protein NIP7
* XP_007603503.1hypotheticalproteinVICG_00050	K02900	102.7	167.3	3.70E-51	large subunit ribosomal protein L27Ae
* XP_007603534.1hypotheticalproteinVICG_00081	K14831	103.43	223.2	5.90E-68	protein MAK16
* XP_007603549.1hypotheticalproteinVICG_00096	K15264	231.47	294.3	1.70E-89	25S rRNA (cytosine2278-C5)-methyltransferase [EC:2.1.1.311]
* XP_007603555.1hypotheticalproteinVICG_00102	K02324	395.57	1437.4	0	DNA polymerase epsilon subunit 1 [EC:2.7.7.7]
* XP_007603586.1hypotheticalproteinVICG_00133	K09467	102.03	124.1	1.30E-37	zinc-finger protein CreA/MIG
* XP_007603650.1hypotheticalproteinVICG_00197	K03136	42.67	132.7	1.80E-40	transcription initiation factor TFIIE subunit alpha
* XP_007603684.1hypotheticalproteinVICG_00231	K11884	123.77	231.6	8.80E-71	RNA-binding protein PNO1
* XP_007603688.1hypotheticalproteinVICG_00235	K14006	287.87	557.8	5.40E-169	protein transport protein SEC23
* XP_007603795.1hypotheticalproteinVICG_00342	K11699	136.37	570.2	1.00E-172	RNA-dependent RNA polymerase [EC:2.7.7.48]
* XP_007603972.1hypotheticalproteinVICG_00520	K10991	48.5	49.7	3.30E-15	DNA repair protein Swi5/Sae3
* XP_007603976.1hypotheticalproteinVICG_00524	K11253	217.8	231.3	2.50E-70	histone H3
* XP_007603998.1hypotheticalproteinVICG_00546	K03038	194	265.2	9.20E-81	26S proteasome regulatory subunit N8
* XP_007604025.1hypotheticalproteinVICG_00573	K06961	67.1	238	1.90E-72	ribosomal RNA assembly protein
* XP_007604027.1hypotheticalproteinVICG_00575	K03142	143.47	217.8	2.10E-66	transcription initiation factor TFIIH subunit 2
* XP_007604045.1hypotheticalproteinVICG_00593	K00981	53.8	184.5	2.80E-56	phosphatidate cytidylyltransferase [EC:2.7.7.41]
* XP_007604066.1hypotheticalproteinVICG_00614	K19613	320.8	322.5	3.80E-98	leucine-rich repeat protein SHOC2
* XP_007604198.1hypotheticalproteinVICG_00747	K11593	439.33	645.6	2.00E-195	eukaryotic translation initiation factor 2C
* XP_007604216.1hypotheticalproteinVICG_00765	K02974	30.53	55.9	3.70E-17	small subunit ribosomal protein S24e
* XP_007604222.1hypotheticalproteinVICG_00771	K02321	119.73	179.4	8.20E-55	DNA polymerase alpha subunit B
* XP_007604260.1hypotheticalproteinVICG_00811	K02912	69.33	150.2	7.50E-46	large subunit ribosomal protein L32e
* XP_007604292.1hypotheticalproteinVICG_00843	K01174	70.97	110.9	6.30E-34	micrococcal nuclease [EC:3.1.31.1]
* XP_007604335.1hypotheticalproteinVICG_00886	K12580	116.73	138	4.40E-42	CCR4-NOT transcription complex subunit 3
* XP_007604339.1hypotheticalproteinVICG_00890	K06072	94.2	260.3	2.20E-79	deoxyhypusine monooxygenase [EC:1.14.99.29]
* XP_007604344.1hypotheticalproteinVICG_00897	K14852	38.47	77.7	9.50E-24	regulator of ribosome biosynthesis
* XP_007604350.1hypotheticalproteinVICG_00903	K01262	246.6	292.9	4.80E-89	Xaa-Pro aminopeptidase [EC:3.4.11.9]
* XP_007604487.1hypotheticalproteinVICG_01041	K14834	108.77	135.5	2.10E-41	nucleolar complex protein 3

# **Gene name**	**KO**	**threshold**	**score**	**E-value**	**KO definition**
* XP_007604512.1hypotheticalproteinVICG_01066	K00600	79.5	663.7	3.30E-201	glycine hydroxymethyltransferase [EC:2.1.2.1]
* XP_007604572.1hypotheticalproteinVICG_01126	K00698	150.03	423.3	2.10E-128	chitin synthase [EC:2.4.1.16]
* XP_007604630.1hypotheticalproteinVICG_01184	K00287	53.83	87.1	1.20E-26	dihydrofolate reductase [EC:1.5.1.3]
* XP_007604760.1hypotheticalproteinVICG_01314	K02925	332.17	587.5	4.00E-178	large subunit ribosomal protein L3e
* XP_007604760.1hypotheticalproteinVICG_01314	K02906	115.77	116.7	1.20E-35	large subunit ribosomal protein L3
* XP_007604773.1hypotheticalproteinVICG_01327	K03357	94.9	95.6	3.30E-29	anaphase-promoting complex subunit 10
* XP_007604813.1hypotheticalproteinVICG_01367	K14007	221.73	355.7	4.10E-108	protein transport protein SEC24
* XP_007604824.1hypotheticalproteinVICG_01378	K21631	59.7	107.7	8.20E-33	Gti1/Pac2 family transcription factor
* XP_007604846.1hypotheticalproteinVICG_01400	K02976	24.73	84.9	5.80E-26	small subunit ribosomal protein S26e
* XP_007605182.1hypotheticalproteinVICG_01737	K02976	24.73	84.9	5.80E-26	small subunit ribosomal protein S26e
* XP_007604961.1hypotheticalproteinVICG_01515	K02934	62.5	74	1.10E-22	large subunit ribosomal protein L6e
* XP_007604991.1hypotheticalproteinVICG_01545	K03025	72.53	195.7	1.40E-59	DNA-directed RNA polymerase III subunit RPC6
* XP_007605048.1hypotheticalproteinVICG_01603	K02542	667.77	810	2.60E-245	DNA replication licensing factor MCM6 [EC:3.6.4.12]
* XP_007605092.1hypotheticalproteinVICG_01647	K14404	67.27	185	3.10E-56	cleavage and polyadenylation specificity factor subunit 4
* XP_007605096.1hypotheticalproteinVICG_01651	K02979	48.23	56.9	2.30E-17	small subunit ribosomal protein S28e
* XP_007605101.1hypotheticalproteinVICG_01656	K03681	98.07	127.7	4.60E-39	exosome complex component RRP40
* XP_007605118.1hypotheticalproteinVICG_01673	K09140	27.83	265.4	1.10E-80	pre-rRNA-processing protein TSR3
* XP_007605132.1hypotheticalproteinVICG_01687	K14326	522.83	603.1	1.10E-182	regulator of nonsense transcripts 1 [EC:3.6.4.-]
* XP_007605148.1hypotheticalproteinVICG_01703	K03165	627.8	727.7	1.90E-220	DNA topoisomerase III [EC:5.6.2.1]
* XP_007605153.1hypotheticalproteinVICG_01708	K02978	30.8	52.7	4.50E-16	small subunit ribosomal protein S27e
* XP_007605168.1hypotheticalproteinVICG_01723	K12598	1016.03	1044.1	0	ATP-dependent RNA helicase DOB1 [EC:3.6.4.13]
* XP_007605236.1hypotheticalproteinVICG_01791	K03014	89.5	114.3	7.50E-35	DNA-directed RNA polymerases I, II, and III subunit RPABC2
* XP_007605241.1hypotheticalproteinVICG_01796	K14788	197.07	206.1	9.30E-63	ribosome biogenesis protein ENP2
* XP_007605243.1hypotheticalproteinVICG_01798	K02541	598.17	732.6	7.50E-222	DNA replication licensing factor MCM3 [EC:3.6.4.12]
* XP_007605348.1hypotheticalproteinVICG_01903	K03514	176.83	227.1	4.20E-69	non-canonical poly(A) RNA polymerase PAPD5/7 [EC:2.7.7.19]
* XP_007605382.1hypotheticalproteinVICG_01937	K02883	33.3	105.8	2.40E-32	large subunit ribosomal protein L18e
* XP_007605513.1hypotheticalproteinVICG_02068	K00728	232.43	393.7	1.50E-119	dolichyl-phosphate-mannose-protein mannosyltransferase [EC:2.4.1.109]
* XP_007605535.1hypotheticalproteinVICG_02090	K19613	320.8	374.1	8.60E-114	leucine-rich repeat protein SHOC2

*Significant scores are highlighted in the output by an asterisk.

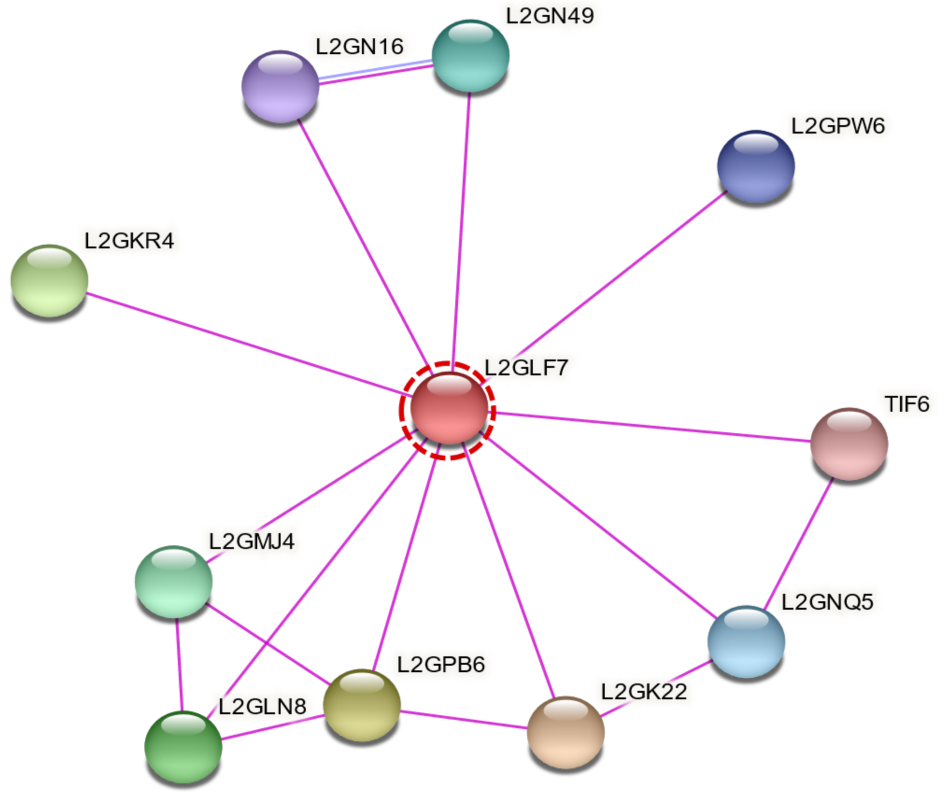

Legend:

Node Type	Node ID	Protein ID
Query node	L2GLF7	Uncharacterized protein
Interacting protein	L2GPB6	Pyr redox 2 domain-containing protein
Interacting protein	L2GKR4	SRP54 domain-containing protein
Interacting protein	L2GLN8	Uncharacterized protein
Interacting protein	L2GK22	Pyruvate kinase
Interacting protein	L2GMJ4	Uncharacterized protein
Interacting protein	L2GN49	Small GTP-binding protein domain protein
Interacting protein	L2GNQ5	SBDS domain-containing protein
Interacting protein	L2GPW6	Uncharacterized protein
Interacting protein	L2GN16	Translation initiation factor aIF-2
Interacting protein	TIF6	Eukaryotic translation initiation factor 6

Figure S1. Protein association network of VICG01314 using STRING highlights close interaction with binding proteins and translation initiation factors.

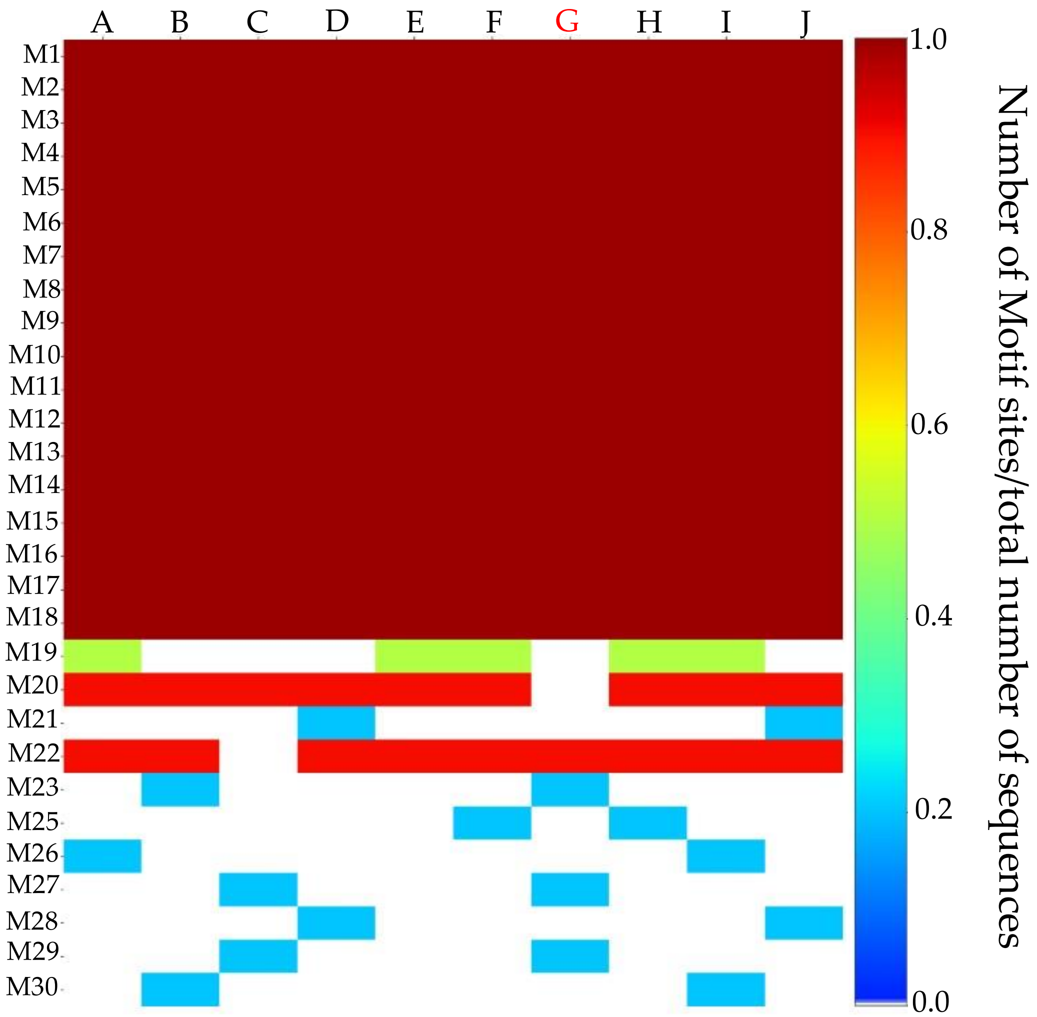

Legend

A.AHW68405.1 | 60S Ribosomal Protein L3-like protein | *Nosema pernyi*

B. OQS55742.1 | Ribosomal Protein L3 | *Enterocytozoon hepatopenaei*

C. ORD97206.1 | Ribosomal Protein L3 | *Hepatospora eriocheir*

D.TBU14344.1 | Ribosomal Protein L3 | *Ordospora colligata*

E. XP_003072614.1 | 60S Ribosomal Protein L3 | *Encephalitozoon intestinalis ATCC 50506*

F. XP_003887974.1 | 60S Ribosomal Protein L3 | *Encephalitozoon hellem ATCC 50504*

G.XP_007604760.1 | Hypothetical Protein VICG01314 | *Vittaforma corneae ATCC 50505*

H.XP_009265254.1 | 60S Ribosomal Protein L3 | *Encephalitozoon romaleae SJ-2008*

I. XP_013162446.1 | 60S Ribosomal Protein L3-like | *Papilio xuthus*

J. XP_014563105.1 | Ribosomal Protein L3 | *Ordospora colligata OC4*

Figure S2. Motif heatmap highlighting 18 highly conserved motifs among the VICG1314 and its homologs. Motif 20 is absent in the HP highlighted in red (G).

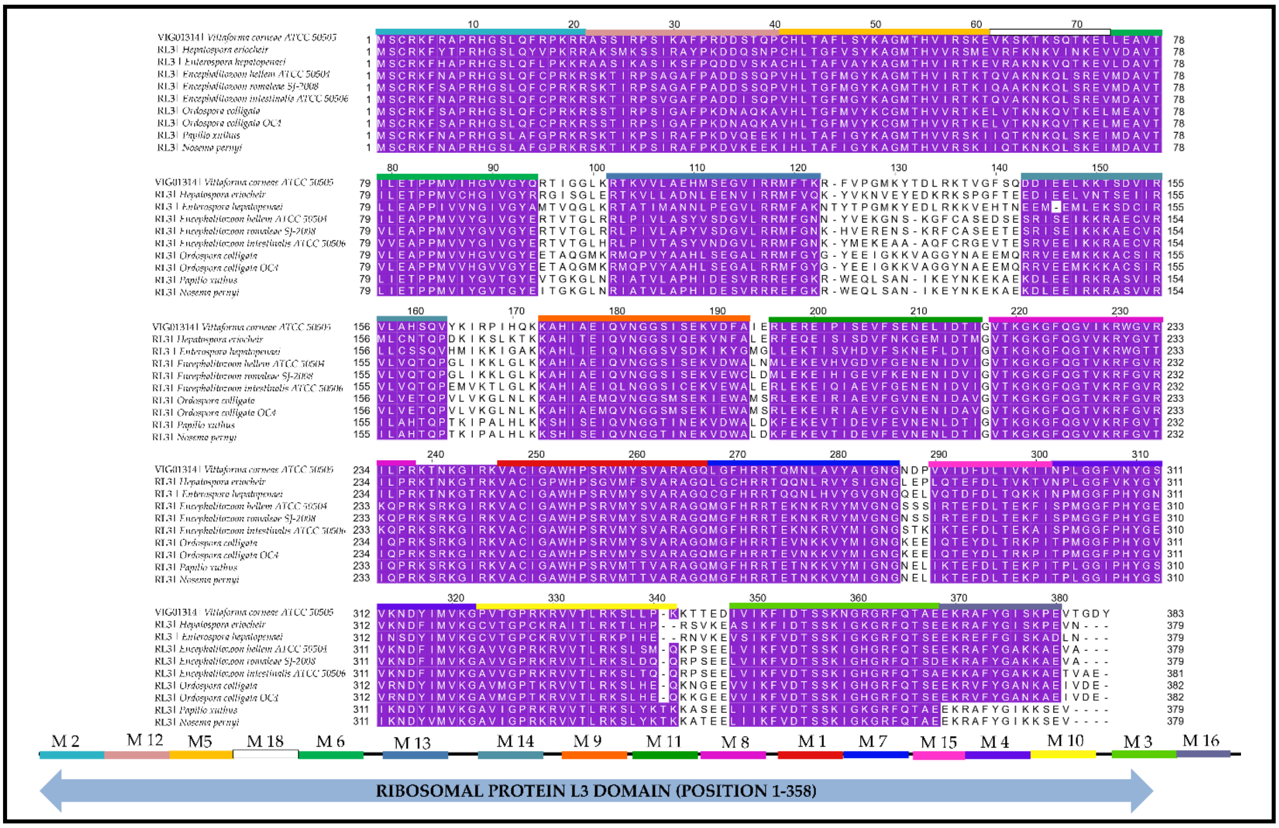

Figure S3: Multiple sequence alignment, motif mapping and a diagrammatic representation of the domain present in HP VICG01314, responsible for regulatory processes.

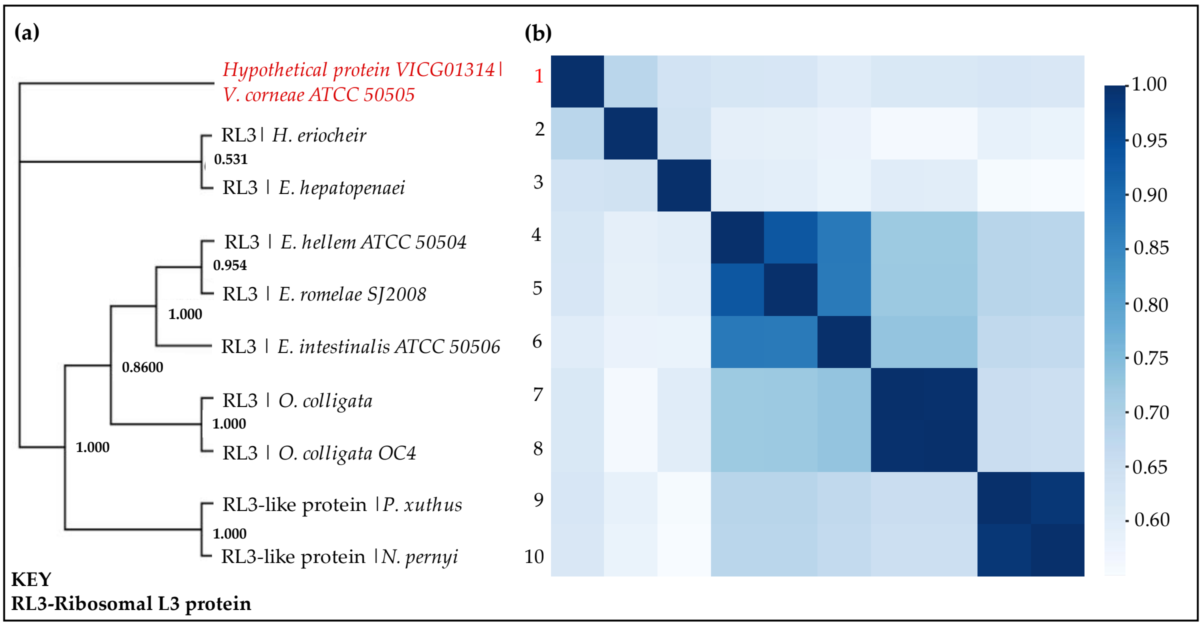

Figure S4: (a). Phylogenetic classification of HP VICG01314 highlighted in red, was done on MEGA X. Phylogeny construction was done using the Maximum Likelihood heuristic search method and the Le-Gascuel 2008 model (+I, parameter=0.11) at 90% site coverage (1000 bootstrap replicates). The initial tree was generated using the default neighbor-joining/BioNJ algorithm. The best tree with the highest log-likelihood of -2737.423 is shown.; (b) Sequence identity heatmap highlighting a pairwise comparison of each of the sequences used in the phylogenetic analysis. A darker color shows a closer identity to respective sequences.

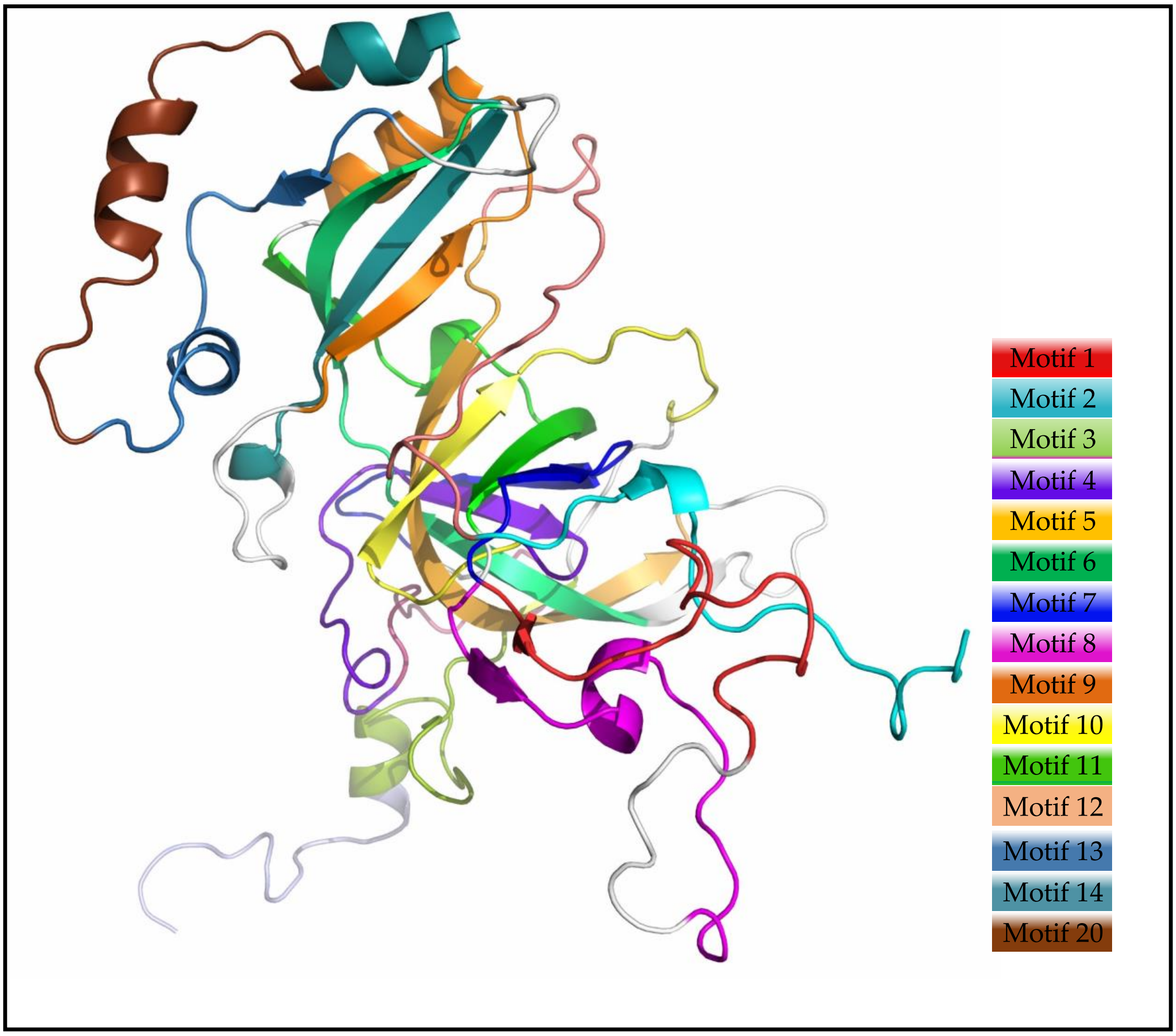

Figure S5: The predicted 3D structure of VICG01314 with motifs mapped in respective positions. Motif numbering is respective of MEME labeling.

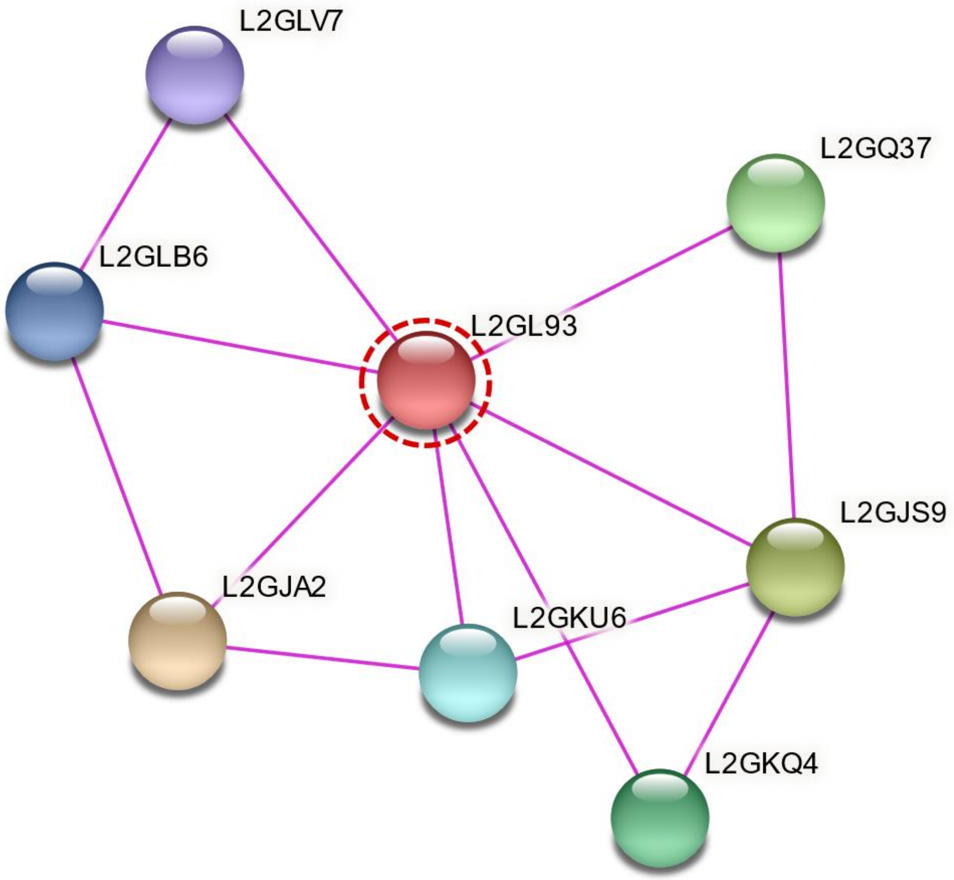

Legend:

Node Type	Node ID	Protein ID
Query node	L2GL93	Uncharacterized protein
Interacting protein	L2GJS9	Uncharacterized protein
Interacting protein	L2GQ37	C2H2-type domain-containing protein
Interacting protein	L2GKQ4	Cyclin N-terminal domain-containing protein
Interacting protein	L2GJA2	SANT domain-containing protein
Interacting protein	L2GKU6	Cyclin domain-containing protein
Interacting protein	L2GLB6	14 3 3 domain-containing protein
Interacting protein	L2GLV7	Ribosomal L7Ae domain -containing protein

Figure S6: Protein-protein interaction analysis of HP VICG01349 using STRING analysis. Close associations with proteins involved in cell cycle processed is indicated.

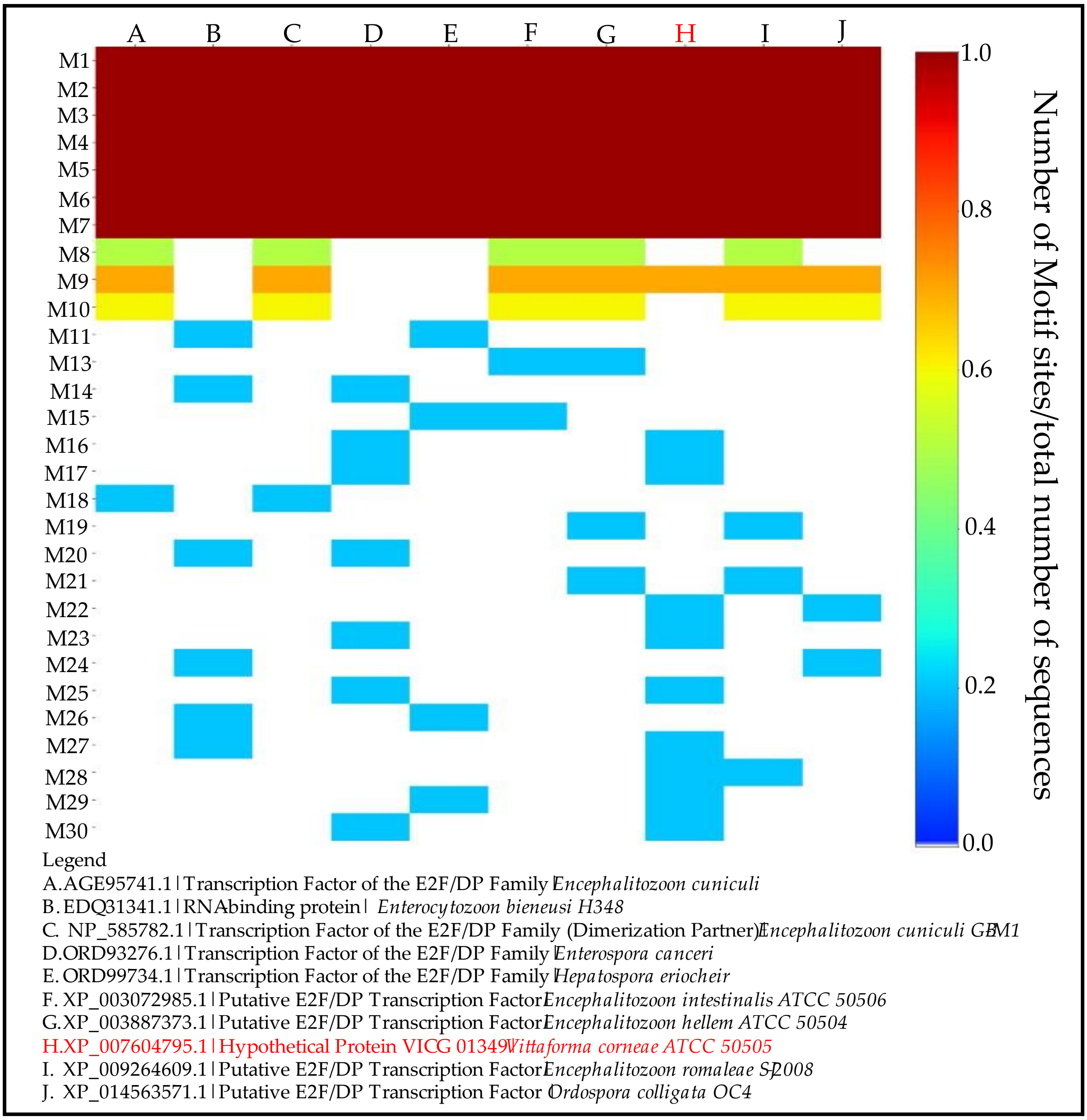

Figure S7: Motif heatmap of VICG1349 showing conservation of 7 motifs across all species. Species and protein IDs are indicated in the legend.

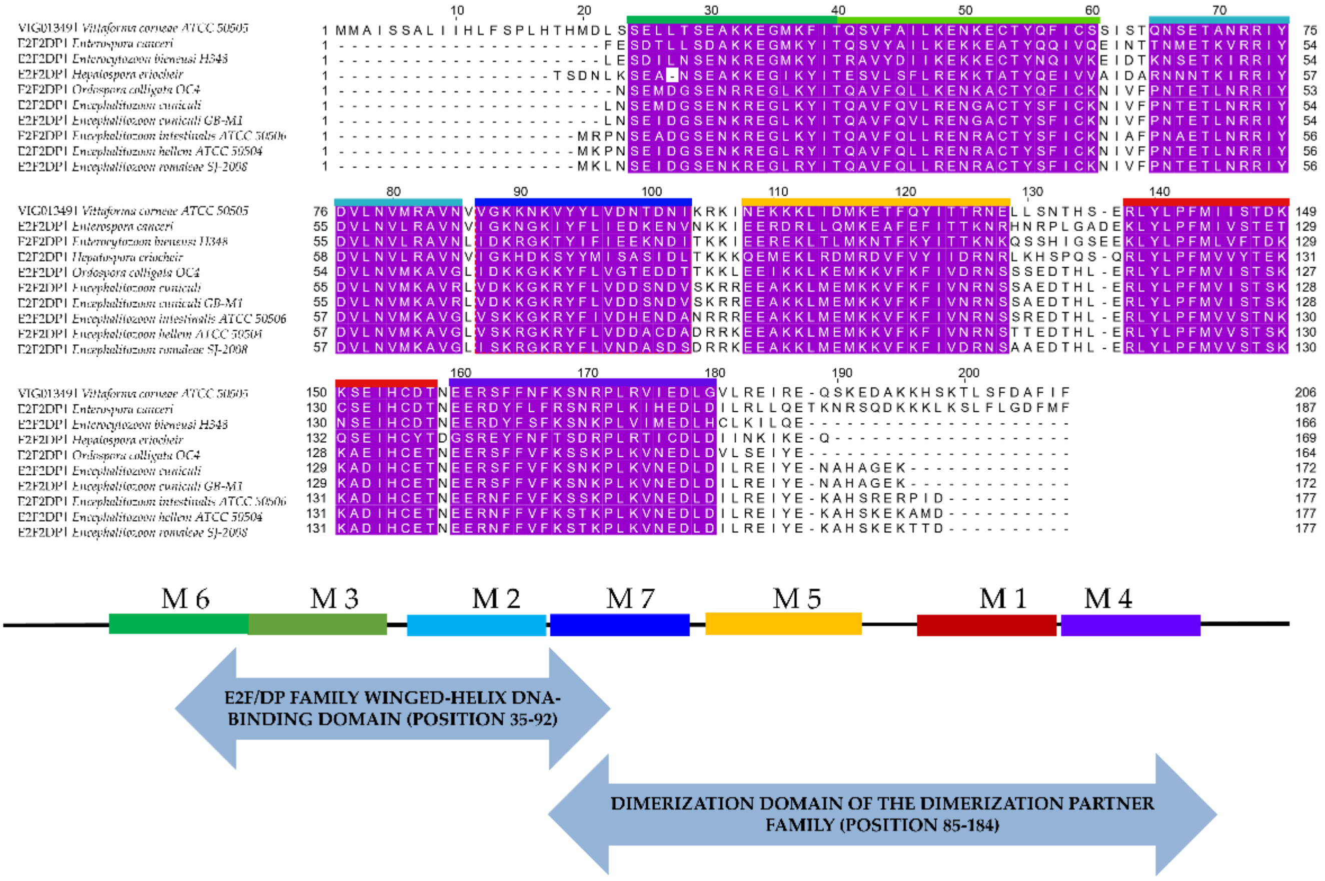

Figure S8: Multiple sequence alignment and motif mapping on VICG01349. Two main domains (the winged-helix DNA-binding domain and the Dimerization domain) are also indicated spanning the region across the sequence as a diagrammatic representation.

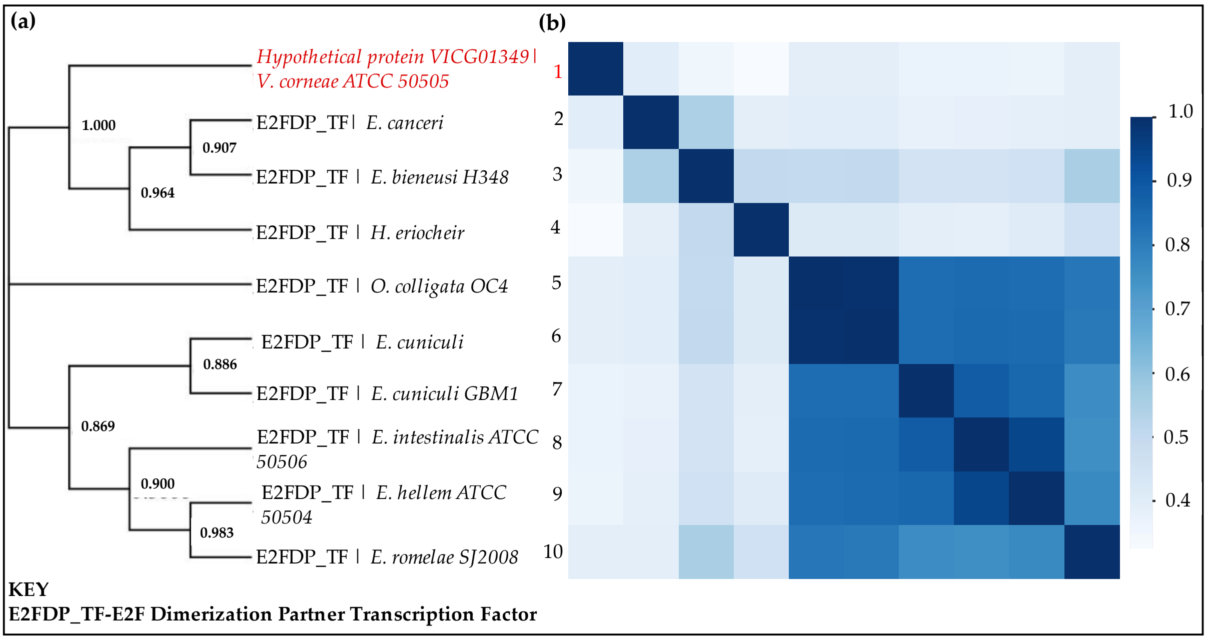

Figure S9: Tree calculations and sequence identity heatmap of VICG01349 and its homologs illustrate the close relationship between proteins within the same clade.

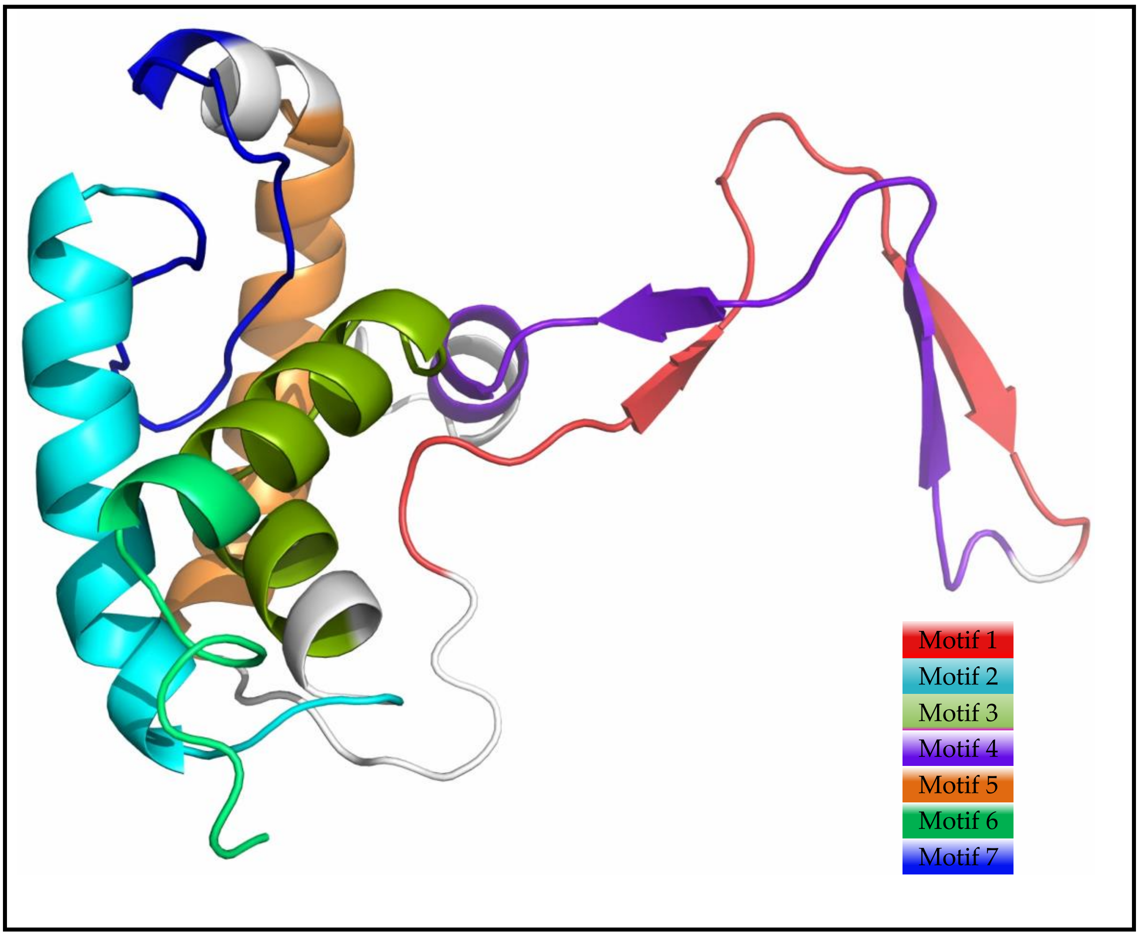

Figure S10: The modeled 3D structure of HP VICG01349 with the seven conserved motifs mapped onto the structure.

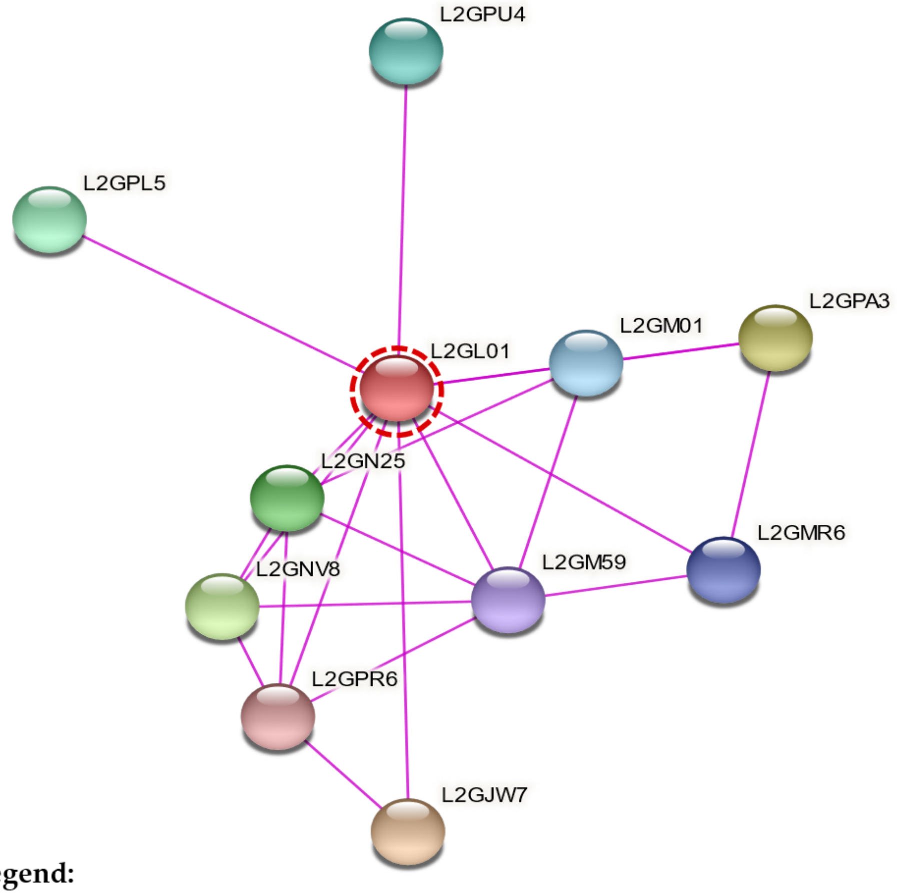

Legend:

Node Type	Node ID	Protein ID
Query node	L2GL01	Uncharacterized protein
Interacting protein	L2GPA3	Uncharacterized protein
Interacting protein	L2GNV8	Nudix hydrolase domain-containing protein
Interacting protein	L2GN25	Uncharacterized protein
Interacting protein	L2GJW7	Small GTP-binding protein domain protein
Interacting protein	L2GPL5	KH dom type 1 domain-containing protein
Interacting protein	L2GPU4	DNA helicase
Interacting protein	L2GM01	Nuclear cap-binding protein subunit 2
Interacting protein	L2GMR6	Uncharacterized protein, argonaute family
Interacting protein	L2GM59	Uncharacterized protein, DEAD box helicase
Interacting protein	L2GPR6	Sm domain-containing protein

Figure S11: STRING analysis of the proteins interacting with VICG01687. The query node indicates the HP in question. Its closest neighbors are proteins from the helicase and argonaute family.

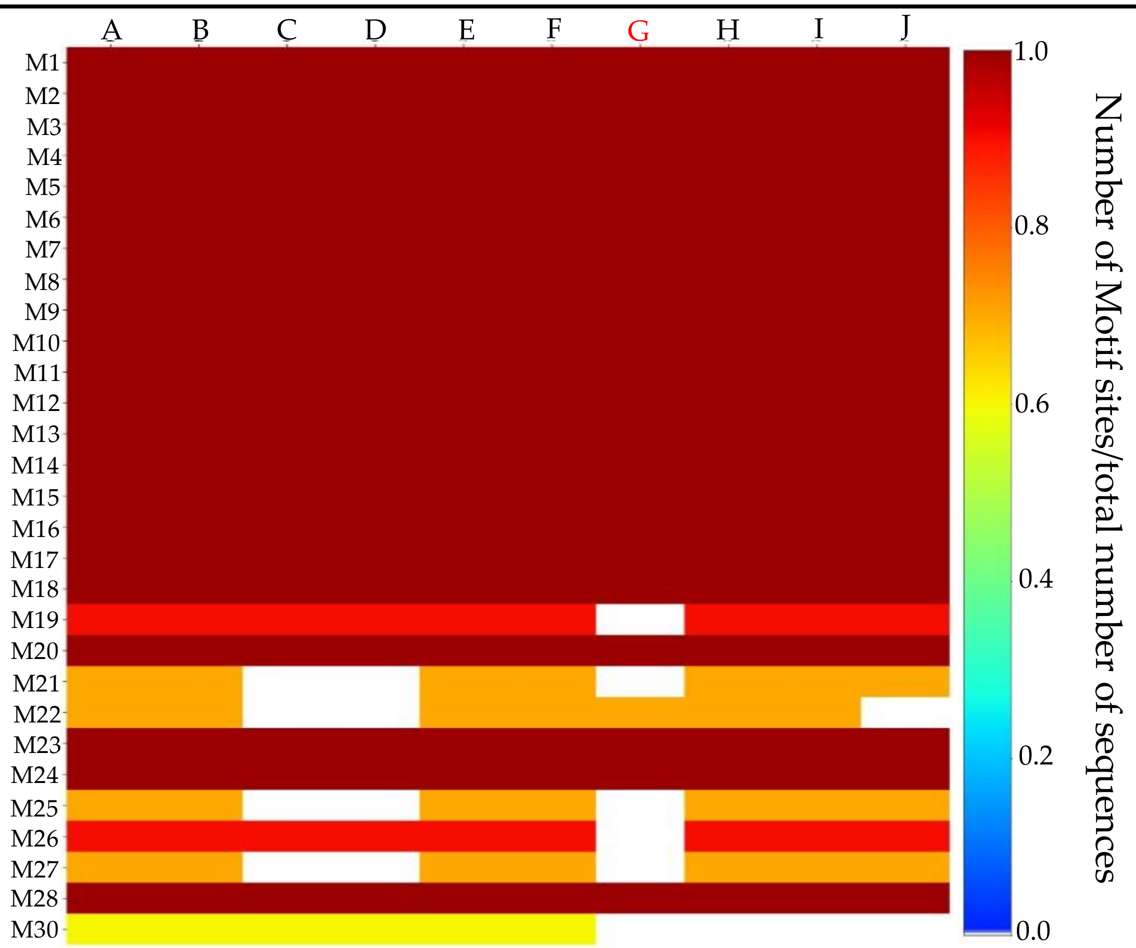

Legend

A. KMV65318.1 | Superfamily I DNA/RNA Helicase | *Encephalitozoon cuniculi EcunIII-L*

B. NP_586281.1 | INVOLVED IN mRNA DECAY CONTROL (DNA2/NAM7 Helicase Family) *Encephalitozoon cuniculi GB-M1*

C. ORD97758.1 | RENT1 *Hepatospora eriocheir*

D. ORD99692.1 | RENT1 *Hepatospora eriocheir*

E. XP_003073846.1 | Putative DNA Helicase | *Encephalitozoon intestinalis ATCC 50506*

F. XP_003888241.1 | Superfamily I DNA/RNA Helicase | *Encephalitozoon hellem ATCC 50504*

G. XP_007605132.1 | Hypothetical Protein VICG 01687 *Vittaforma corneae ATCC 50505*

H. XP_013162264.1 | ATP-dependent Helicase UPF1-like | *Papilio xuthus*

I. XP_013175578.1 | ATP-dependent Helicase UPF1-like | *Papilio xuthus*

J. XP_024330903.1 | Regulator of Nonsense Transcripts 1-like Isoform 2 | *Nosema ceranae*

Figure S12: Motif analysis heatmap of the protein VICG01687, highlighted in red. A large number of motifs were conserved among these proteins. Motifs 19 and 26, conserved in other species appear to be absent in this HP.

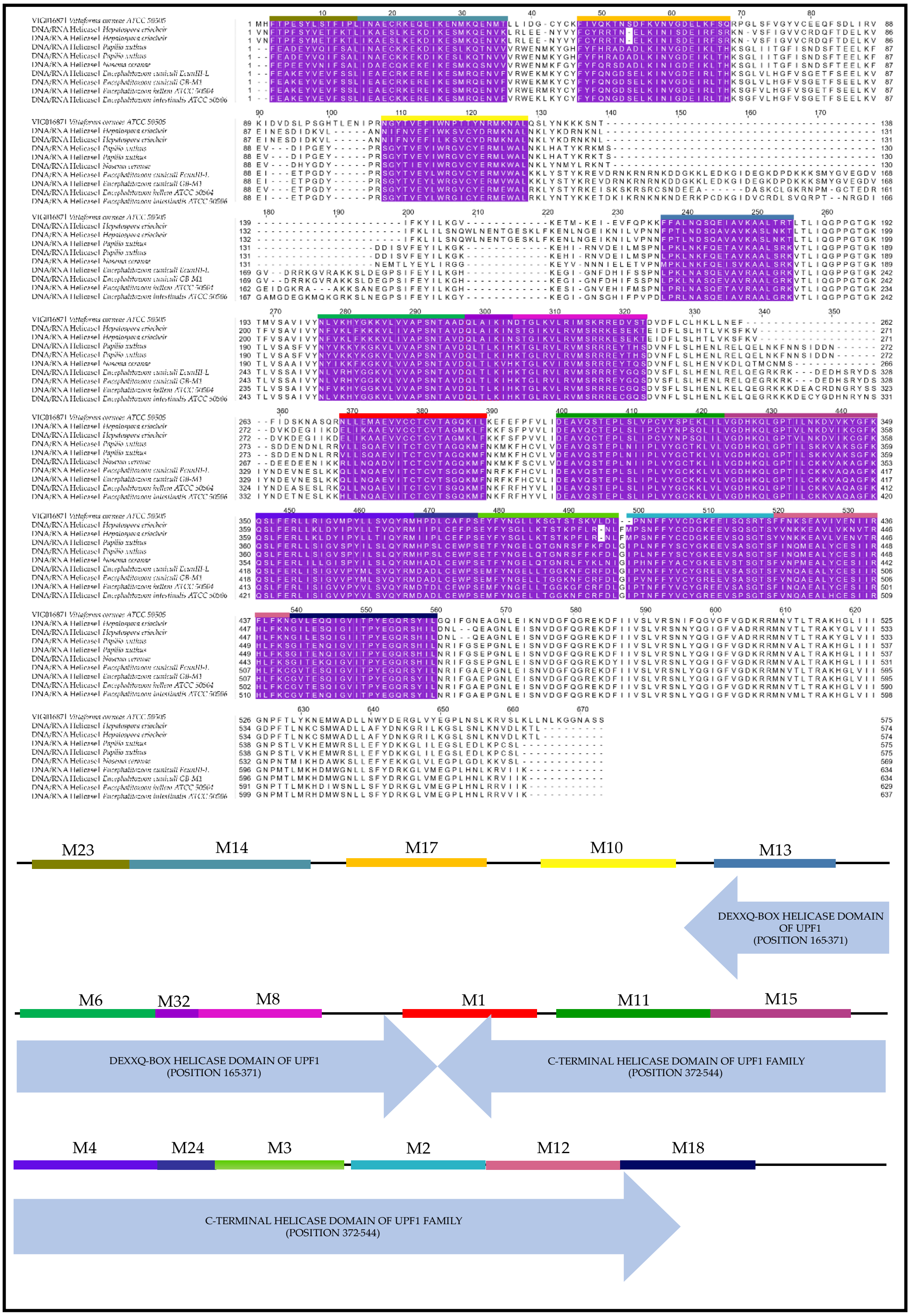

Figure S13. Multiple sequence alignment and motif mapping of VICG01687. Two main domains are mapped onto the diagrammatic representation of the one-dimensional sequence illustrated below the alignment.

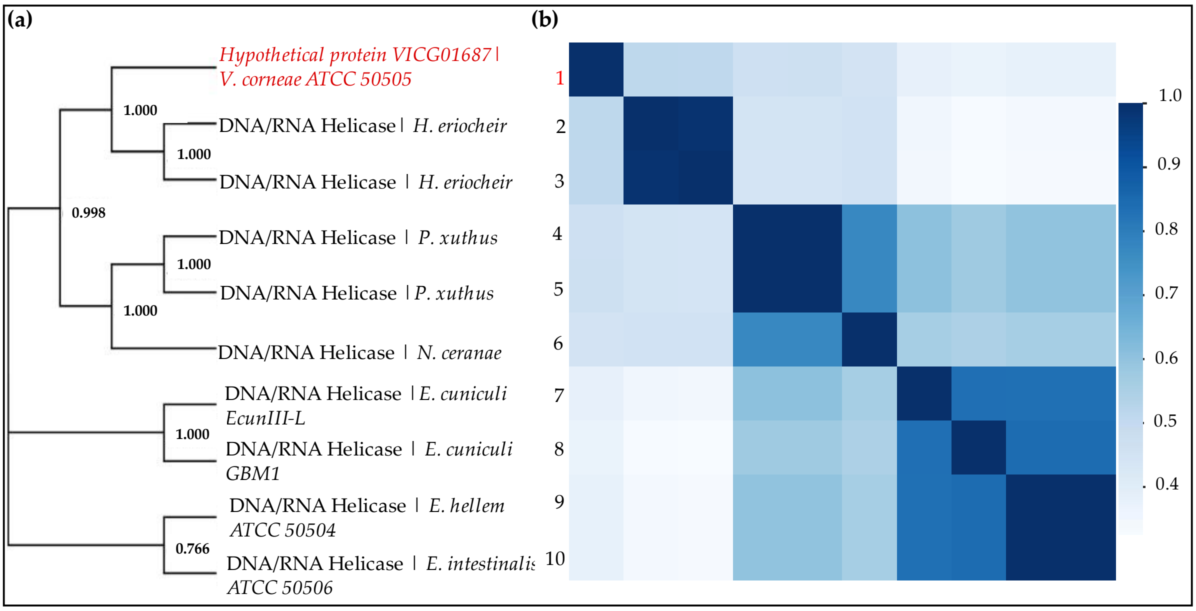

Figure S14: Phylogenetic analysis and pairwise identity calculations of VICG01687 and its homologs. Two distinct clusters are observed.

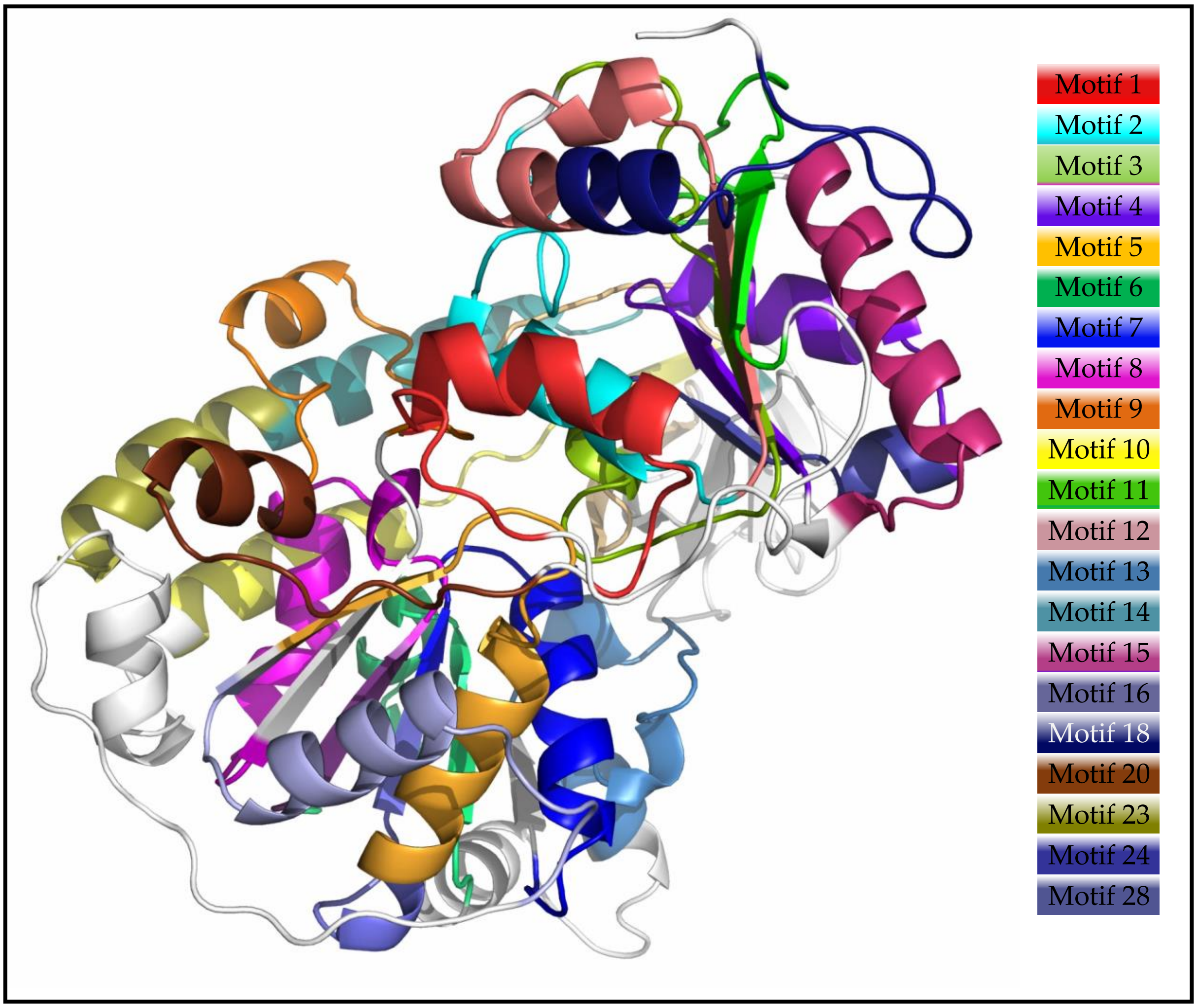

Figure S15: The 3D model of VICG01687 as predicted using Modeller. Motif mapping is based on the most conserved motif identified using MEME.

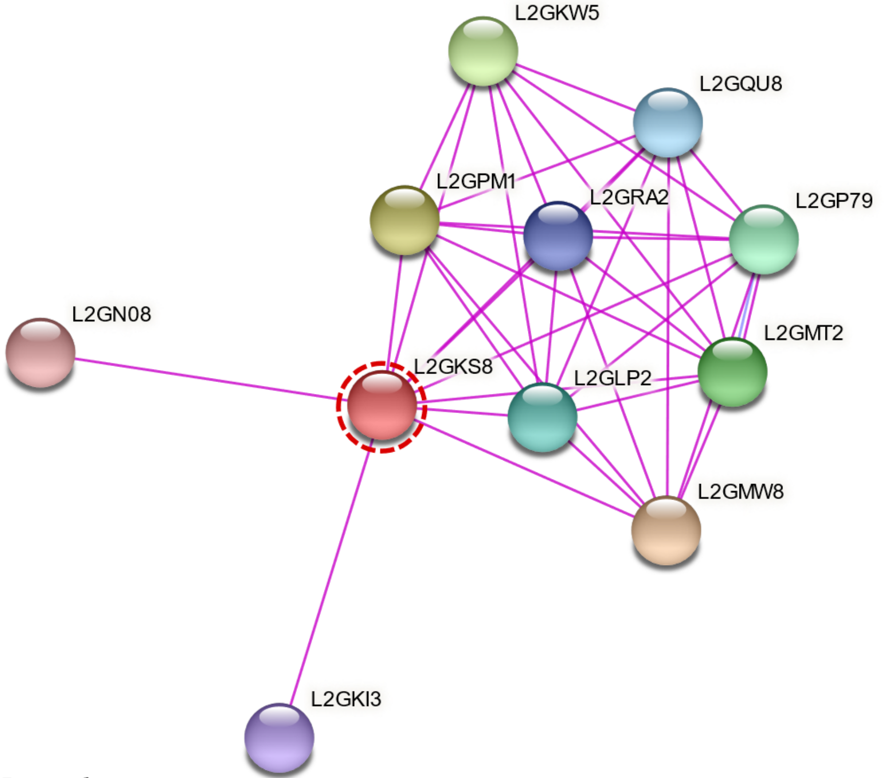

Legend:

Node Type	Node ID	Protein ID
Query node	L2GKS8	Uncharacterized protein
Interacting protein	L2GPM1	Uncharacterized protein
Interacting protein	L2GKW5	Ribosomal S13 N-domain-containing protein
Interacting protein	L2GMT2	WD repeats region domain-containing protein
Interacting protein	L2GMW8	VacB and RNase II family 3'-5' exoribonuclease
Interacting protein	L2GP79	Uncharacterized protein
Interacting protein	L2GLP2	Uncharacterized protein
Interacting protein	L2GQU8	Ribosomal protein S23 (S12)
Interacting protein	L2GRA2	Ribosomal S4e domain-containing protein
Interacting protein	L2GKI3	Poly(A) polymerase
Interacting protein	L2GN08	Uncharacterized protein

Figure S16: Protein interaction network analysis of VICG01723 (query node in red) showing close association with ribosomal proteins.

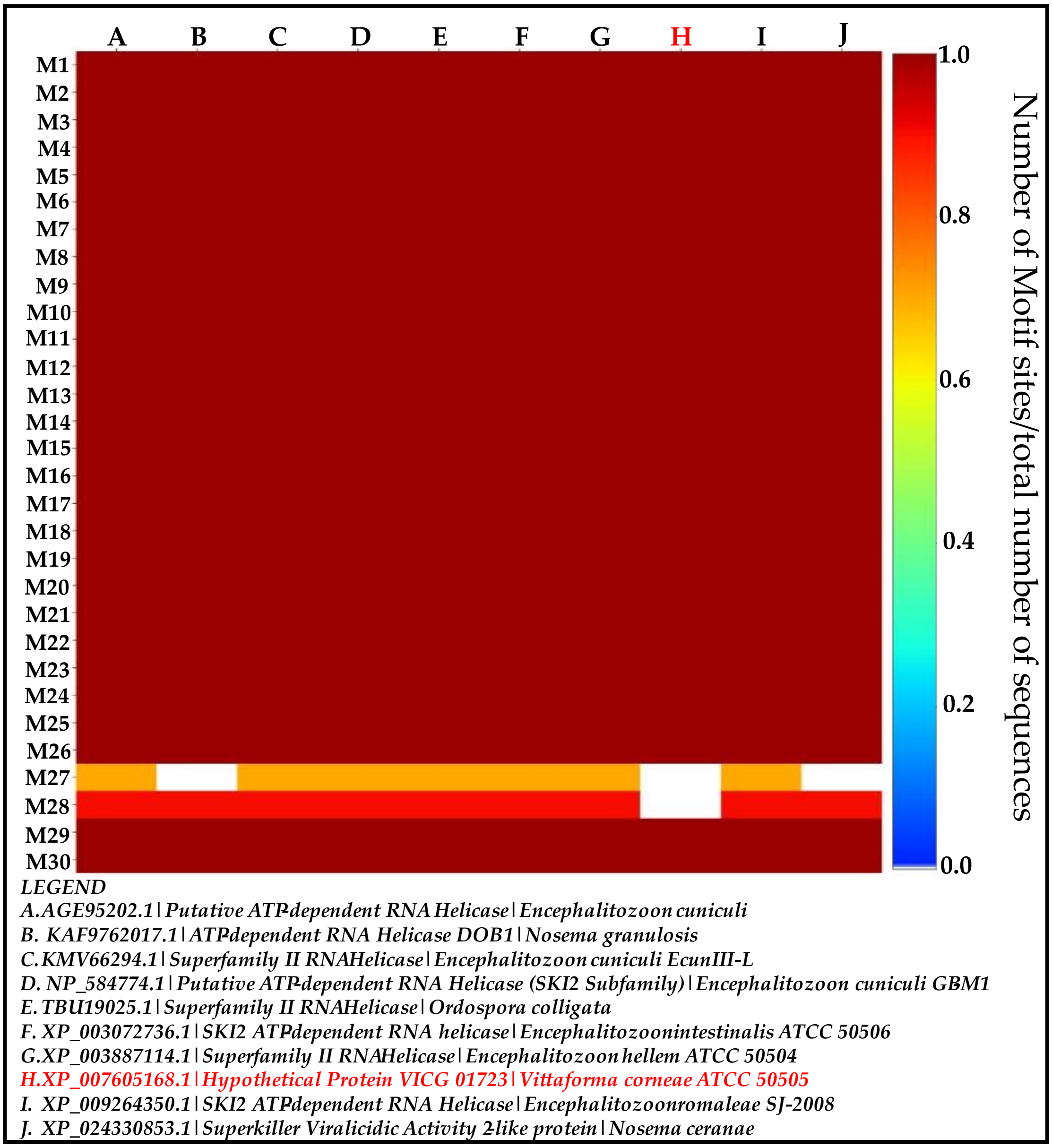

Figure S17: Motif analysis heatmap of VICG01723 shows a high number of conserved motifs. Twenty-eight of the 30 motifs were highly conserved with one motif (Motif 28) seemingly only missing from the HP.

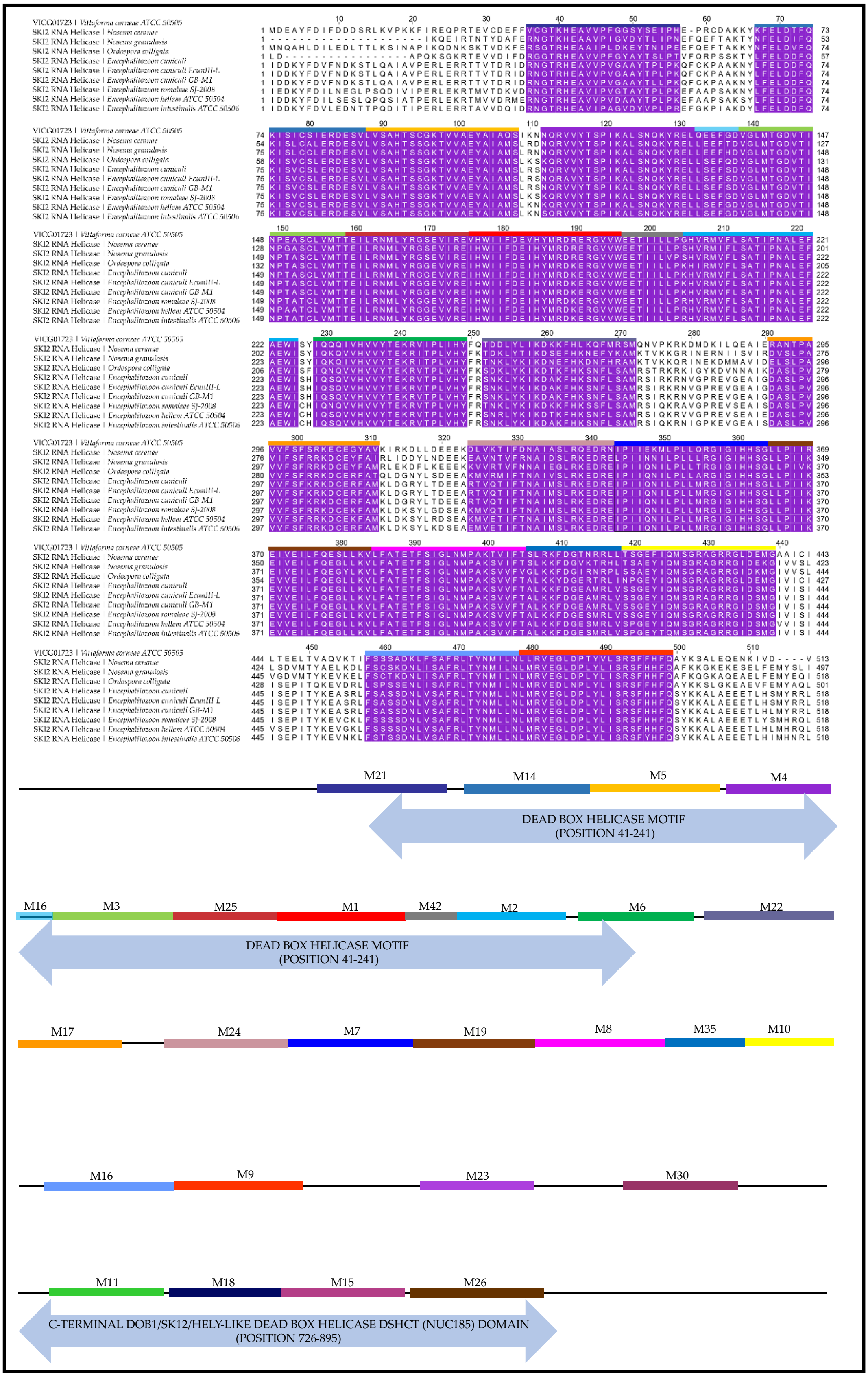

Figure S18: Multiple sequence alignment of the HP VICG01723 with motif mapping respective to MEME results. An N-terminal domain (DEAD-BOX HELICASE) and a C-terminal domain are highlighted in the sequence.

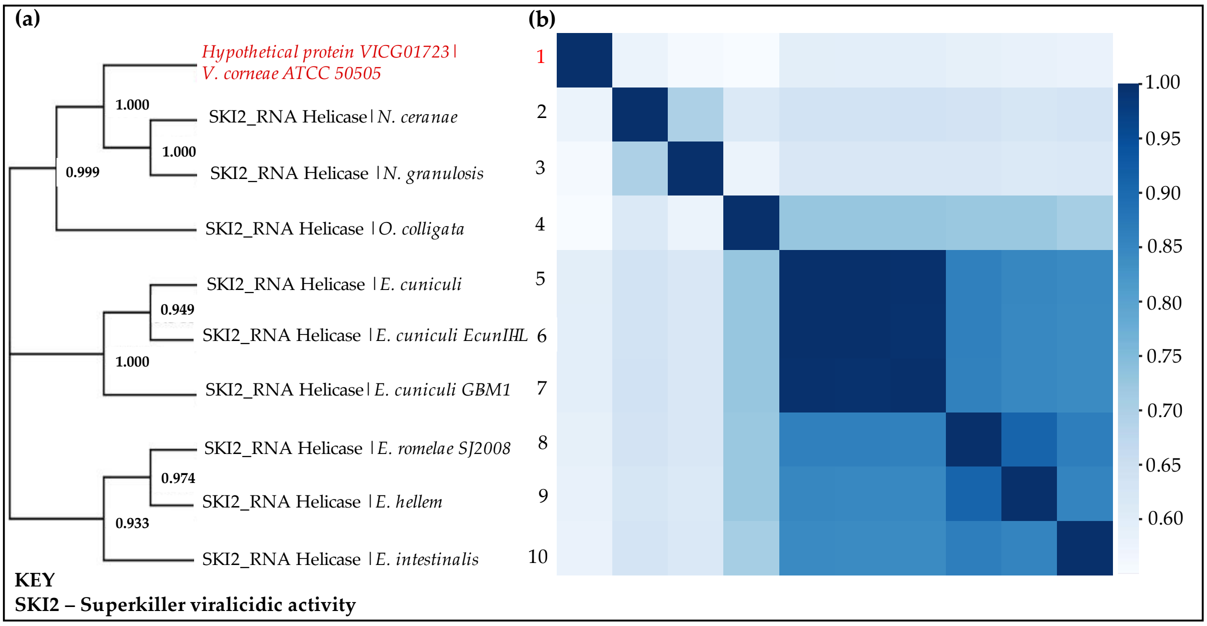

Figure S19: (**a**) Phylogenetic classification of HP VICG01723 (UniProt ID: L2GKS8). Tree calculations were done using the Maximum Likelihood statistical method, with 1000 bootstrap replicates. The Le-Gascuel (2008) LG model was used at 90% site coverage. Gamma site distribution with 5 discrete categories (+G, parameter= - 0.404, +I, parameter= 0.053). The tree with the highest log likelihood (-8438.537) is shown; (**b**) Pairwise sequence calculations highlight the sequence identity values among the homologous species.

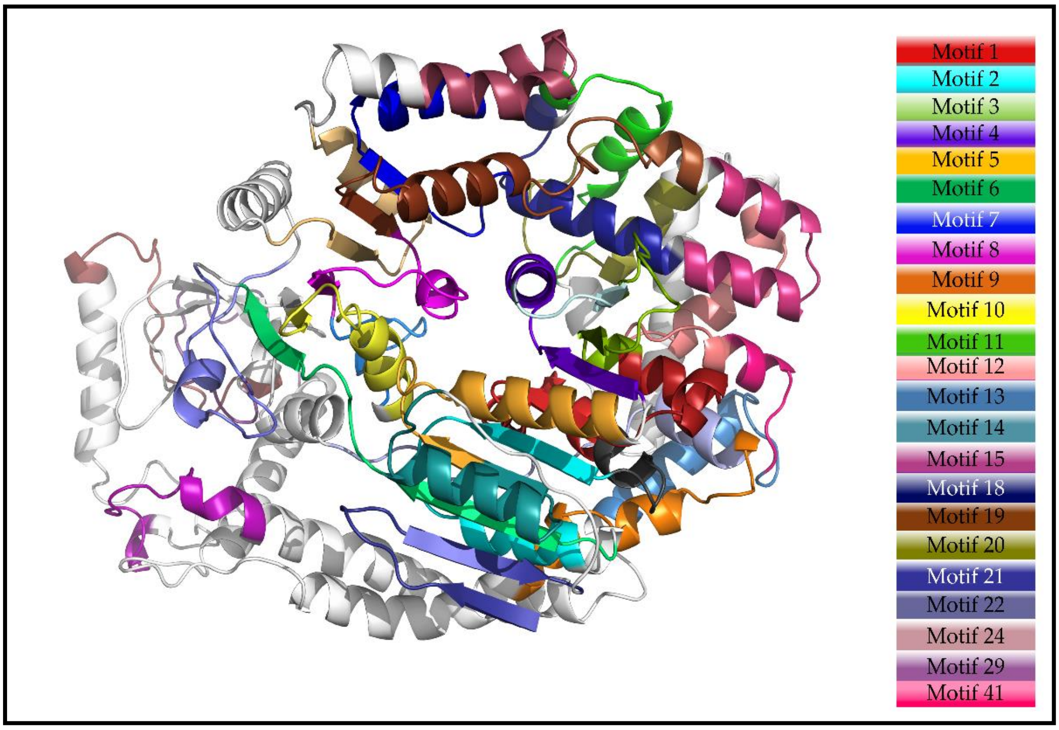

Figure S20: The 3D modeled structure of VICG01723 with motif mapping.

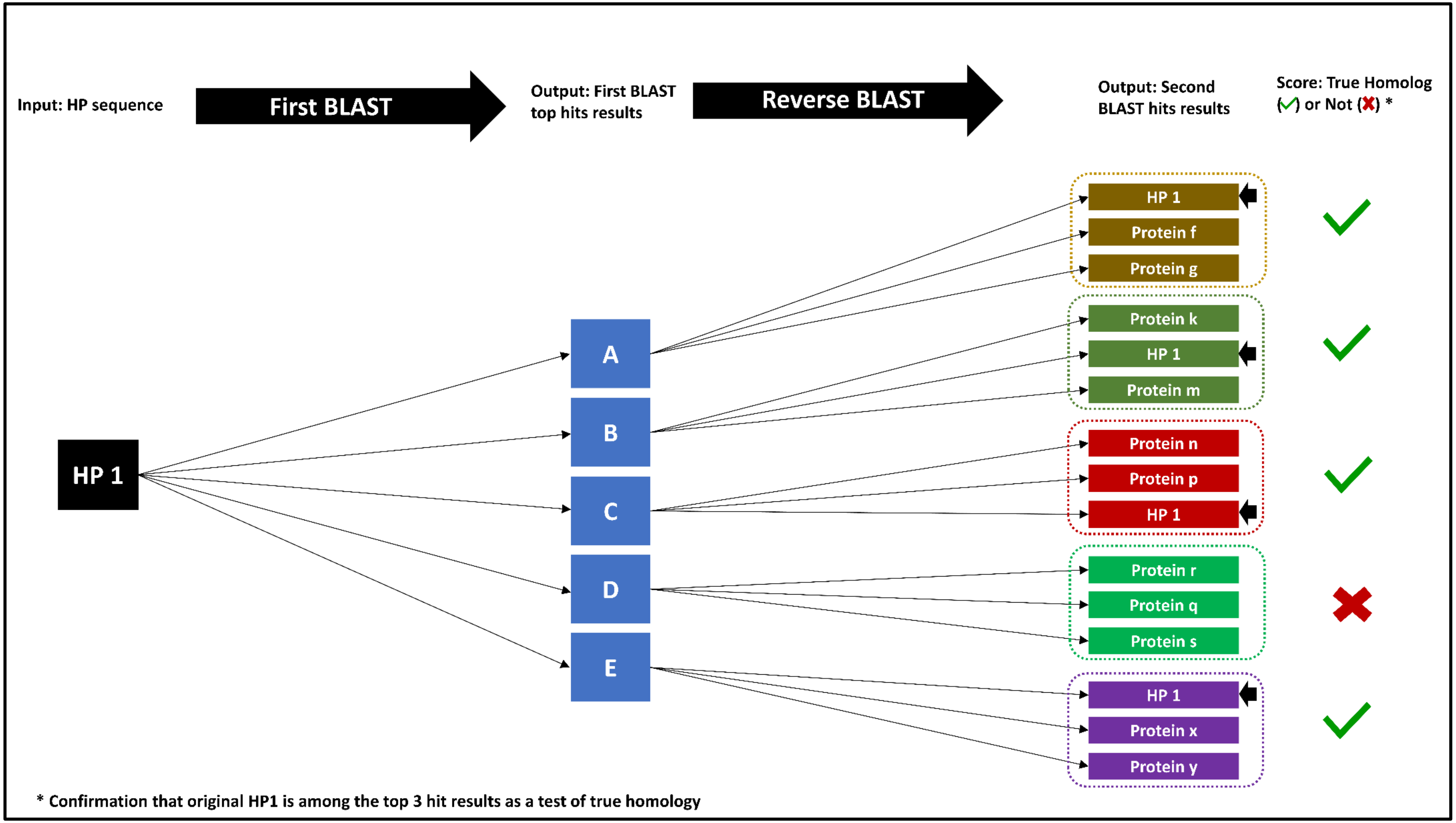

Figure S21: A schematic diagram demonstrating the reverse BLAST workflow used to classify true homologs.

Table S6: 3D structure validation scores

MODEL	PROSA Z-SCORE	VERIFY3D %Residues with 3D-1D score >=0.2	PROCHECK Residues in most favoured regions	PROCHECK Residues in additional allowed regions	PROCHECK Residues in generously allowed regions	PROCHECK Residues in disallowed regions	DOPE Z-SCORE MODELLER	QMEAN Z-SCORE
VICG00012	-7.59	86.61%	90.6%	6.9%	2.0%	0.5%	-0.781	-3.84
VICG01314	-5.93	71.28%	89.0%	9.5%	0.6%	0.9%	0.732	-2.88
VICG01349	-2.92	50.96%	86.0%	11.3%	2.7%	0.0%	0.457	-3.95
VICG01687	-9.68	80.04%	92.8%	4.8%	1.2%	1.2%	-0.876	-3.32
VICG01723	-11.11	69.22%	89.5%	8.1%	1.1%	1.2%	-0.115	-4.06

References

1. Johnson, M.; Zaretskaya, I.; Raytselis, Y.; Merezhuk, Y.; McGinnis, S.; Madden, T.L. NCBI BLAST: a better web interface. *Nucleic Acids Res.* **2008**, *36*, 5–9. https://doi.org/10.1093/nar/gkn201.
2. Aurrecoechea, C.; Barreto, A.; Brestelli, J.; Brunk, B.P.; Caler, E. V.; Fischer, S.; Gajria, B.; Gao, X.; Gingle, A.; Grant, G.; et al. AmoebaDB and MicrosporidiaDB: Functional genomic resources for Amoebozoa and Microsporidia species. *Nucleic Acids Res.* **2011**, *39*, 612–619. https://doi.org/10.1093/nar/gkq1006.
3. Bateman, A.; Martin, M.J.; Orchard, S.; Magrane, M.; Agivetova, R.; Ahmad, S.; Alpi, E.; Bowler-Barnett, E.H.; Britto, R.; Bursteinas, B.; et al. UniProt: The universal protein knowledgebase in 2021. *Nucleic Acids Res.* **2021**, *49*, D480–D489. https://doi.org/10.1093/nar/gkaa1100.
4. Rossum, G. Van; Drake, F.L. Python Tutorial. *Python Softw. Found.* **2017**, *42*, 1–122.
5. Silaparasetty, N. Python Programming in Jupyter Notebook. In *Machine Learning Concepts with Python and the Jupyter Notebook Environment*; Apress: Berkeley, **2020**; pp. 119–145 ISBN 978-1-4842-5967-2.
6. Mistry, J.; Chuguransky, S.; Williams, L.; Qureshi, M.; Salazar, G.A.; Sonnhammer, E.L.L.; Tosatto, S.C.E.; Paladin, L.; Raj, S.; Richardson, L.J.; et al. Pfam: The protein families database in 2021. *Nucleic Acids Res.* **2021**, *49*, D412–D419. https://doi.org/10.1093/nar/gkaa913.
7. Blum, M.; Chang, H.Y.; Chuguransky, S.; Grego, T.; Kandasaamy, S.; Mitchell, A.; Nuka, G.; Paysan-Lafosse, T.; Qureshi, M.; Raj, S.; et al. The InterPro protein families and domains database: 20 years on. *Nucleic Acids Res.* **2021**, *49*, D344–D354. https://doi.org/10.1093/nar/gkaa977.
8. Potter, S.C.; Luciani, A.; Eddy, S.R.; Park, Y.; Lopez, R.; Finn, R.D. HMMER web server: 2018 update. *Nucleic Acids Res.* **2018**, *46*, W200–W204. https://doi.org/10.1093/nar/gky448.
9. Mi, H.; Ebert, D.; Muruganujan, A.; Mills, C.; Albou, L.P.; Mushayamaha, T.; Thomas, P.D. PANTHER version 16: A revised family classification, tree-based classification tool, enhancer regions and extensive API. *Nucleic Acids Res.* **2021**, *49*, D394–D403. https://doi.org/10.1093/nar/gkaa1106.
10. Attwood, T.K. The PRINTS database: a resource for identification of protein families. *Brief. Bioinform.* **2002**, *3*, 252–263. https://doi.org/10.1093/bib/3.3.252.
11. Wu, C.H.; Nikolskaya, A.; Huang, H.; Yeh, L.S.; Natale, D.A.; Vinayaka, C.R.; Hu, Z.Z.; Mazumder, R.; Kumar, S.; Kourtesis, P.; et al. PIRSF: Family classification system at the Protein Information Resource. *Nucleic Acids Res.* **2004**, *32*, 112–114. . https://doi.org/10.1093/nar/gkh097.
12. Kumar, S.; Stecher, G.; Li, M.; Knyaz, C.; Tamura, K. MEGA X: Molecular evolutionary genetics analysis across computing platforms. *Mol. Biol. Evol.* **2018**, *35*, 1547–1549. https://doi.org/10.1093/molbev/msy096.
13. Kumar, S.; Stecher, G.; Peterson, D.; Tamura, K. MEGA-CC: Computing core of molecular evolutionary genetics analysis program for automated and iterative data analysis. *Bioinformatics* **2012**, *28*, 2685–2686. https://doi.org/10.1093/bioinformatics/bts507.
14. Edgar, R.C.; Drive, R.M.; Valley, M. MUSCLE: Multiple sequence alignment with high accuracy and high throughput. *Nucleic Acids Res.* **2004**, *32*, 1792–1797. https://doi.org/10.1093/nar/gkh340.
15. Huson, D.H.; Richter, D.C.; Rausch, C.; Dezulian, T.; Franz, M.; Rupp, R. Dendroscope: An interactive viewer for large phylogenetic trees. *BMC Bioinformatics* **2007**, *8*, 1–6. https://doi.org/10.1186/1471-2105-8-460.
16. Bailey, T.L.; Johnson, J.; Grant, C.E.; Noble, W.S. The MEME Suite. *Nucleic Acids Res.* **2015**, *43*, W39–W49. https://doi.org/10.1093/nar/gkv416.
17. de Castro, E.; Sigrist, C.J.A.; Gattiker, A.; Bulliard, V.; Langendijk-Genevaux, P.S.; Gasteiger, E.; Bairoch, A.; Hulo, N. ScanProsite: Detection of PROSITE signature matches and ProRule-

associated functional and structural residues in proteins. *Nucleic Acids Res.* **2006**, *34*, 362–365. https://doi.org/10.1093/nar/gkl124.
18. Lu, S.; Wang, J.; Chitsaz, F.; Derbyshire, M.K.; Geer, R.C.; Gonzales, N.R.; Gwadz, M.; Hurwitz, D.I.; Marchler, G.H.; Song, J.S.; et al. CDD/SPARCLE: The conserved domain database in 2020. *Nucleic Acids Res.* **2020**, *48*, D265–D268. https://doi.org/10.1093/nar/gkz991.
19. Letunic, I.; Khedkar, S.; Bork, P. SMART: Recent updates, new developments and status in 2020. *Nucleic Acids Res.* **2021**, *49*, D458–D460. https://doi.org/10.1093/nar/gkaa937.
20. Gasteiger, E.; Hoogland, C.; Gattiker, A.; Duvaud, S.; Wilkins, M.R.; Appel, R.D.; Bairoch, A. The Proteomics Protocols Handbook. *Proteomics Protoc. Handb.* **2005**, 571–608. https://doi.org/10.1385/1592598900.
21. Sonnhammer, E.L.L.; Krogh, A. A hidden Markov model for predicting transmembrane helices in protein sequence. *Sixth Int. Conf. Intell. Syst. Mol. Biol.* **2008**, 8.
22. Armenteros, J.J.A.; Salvatore, M.; Emanuelsson, O.; Winther, O.; Von Heijne, G.; Elofsson, A.; Nielsen, H. Detecting sequence signals in targeting peptides using deep learning. *Life Sci. Alliance* **2019**, *2*, 1–14. https://doi.org/10.26508/lsa.201900429.
23. Almagro Armenteros, J.J.; Tsirigos, K.D.; Sønderby, C.K.; Petersen, T.N.; Winther, O.; Brunak, S.; von Heijne, G.; Nielsen, H. SignalP 5.0 improves signal peptide predictions using deep neural networks. *Nat. Biotechnol.* **2019**, *37*, 420–423. https://doi.org/10.1038/s41587-019-0036-z.
24. Käll, L.; Krogh, A.; Sonnhammer, E.L.L. A combined transmembrane topology and signal peptide prediction method. *J. Mol. Biol.* **2004**, *338*, 1027–1036. https://doi.org/10.1016/j.jmb.2004.03.016.
25. Szklarczyk, D.; Gable, A.L.; Nastou, K.C.; Lyon, D.; Kirsch, R.; Pyysalo, S.; Doncheva, N.T.; Legeay, M.; Fang, T.; Bork, P.; et al. The STRING database in 2021: Customizable protein-protein networks, and functional characterization of user-uploaded gene/measurement sets. *Nucleic Acids Res.* **2021**, *49*, D605–D612. https://doi.org/10.1093/nar/gkaa1074.
26. Kanehisa, M.; Sato, Y.; Kawashima, M.; Furumichi, M.; Tanabe, M. KEGG as a reference resource for gene and protein annotation. *Nucleic Acids Res.* **2016**, *44*, D457–D462. https://doi.org/10.1093/nar/gkv1070.
27. Hatherley, R.; Brown, D.K.; Glenister, M.; Bishop, Ö.T. PRIMO: An interactive homology modeling pipeline. *PLoS One* **2016**, *11*, 1–20. https://doi.org/10.1371/journal.pone.0166698.
28. Söding, J.; Biegert, A.; Lupas, A.N. The HHpred interactive server for protein homology detection and structure prediction. *Nucleic Acids Res.* **2005**, *33*, 244–248. https://doi.org/10.1093/nar/gki408.
29. Wiederstein, M.; Sippl, M.J. ProSA-web: Interactive web service for the recognition of errors in three-dimensional structures of proteins. *Nucleic Acids Res.* **2007**, *35*, 407–410. https://doi.org/10.1093/nar/gkm290.
30. Luthy, R.; Bowei, J.; Einsenberg, D. Verify3D: Assessment of protein models with three-dimensional profiles. *Methods Enzymol.* **1997**, *277*, 396–404. https://doi.org/10.1016/S0076-6879(97)77022-8.
31. Pontius, J.; Richelle, J.; Wodak, S.J. Deviations from Standard Atomic Volumes as a Quality Measure for Protein Crystal Structures. *J. Mol. Biol.* **1996**, *264*, 121–136. https://doi.org/10.1006/jmbi.1996.0628.
32. Benkert, P.; Biasini, M.; Schwede, T. Toward the estimation of the absolute quality of individual protein structure models. *Bioinformatics* **2011**, *27*, 343–350. https://doi.org/10.1093/bioinformatics/btq662.
33. Waterhouse, A.; Bertoni, M.; Bienert, S.; Studer, G.; Tauriello, G.; Gumienny, R.; Heer, F.T.; De Beer, T.A.P.; Rempfer, C.; Bordoli, L.; et al. SWISS-MODEL: Homology modelling of protein structures and complexes. *Nucleic Acids Res.* **2018**, *46*, W296–W303. https://doi.org/10.1093/nar/gky427.
